# Tracking Prenucleation Molecular Clustering of Salicylamide in Organic Solvents

**DOI:** 10.1021/acs.cgd.4c00507

**Published:** 2024-06-21

**Authors:** Shubhangi Kakkar, Shayon Bhattacharya, Pierre-André Cazade, Damien Thompson, Åke Rasmuson

**Affiliations:** † Department of Chemical Sciences, Bernal Institute, University of Limerick, Limerick V94 T9PX, Ireland; ‡ Department of Physics, Bernal Institute, University of Limerick, Limerick V94 T9PX, Ireland; § Department of Chemical Engineering and Technology, KTH Royal Institute of Technology, Stockholm SE-10044, Sweden

## Abstract

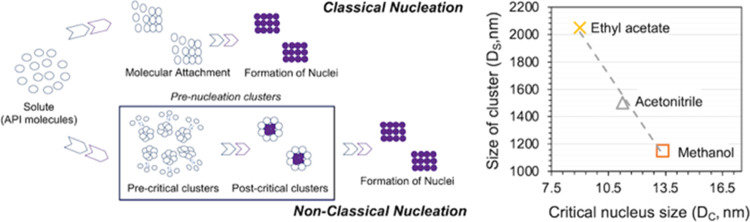

Crystal nucleation shapes the structure and product size distribution of solid-state pharmaceuticals and is seeded by early-stage molecular self-assemblies formed in host solution. Here, molecular clustering of salicylamide in ethyl acetate, methanol, and acetonitrile was investigated using photon correlation spectroscopy. Cluster size steadily increased over 3 days and with concentration across the range from undersaturated to supersaturated solutions. Solute concentration normalized by solubility provided more sensitive characterization of molecular-level conditions than concentration alone. In saturated solution, cluster size is independent of solvent, while at equal supersaturation, solvent-dependent cluster size increases as methanol < acetonitrile < ethyl acetate, commensurate with increasing nucleation propensity. In ethyl acetate, with largest prenucleation clusters, the driving force required for nucleation is lowest, compared to methanol with smallest clusters and highest driving force. To understand solvent–solute effects, we performed IR spectroscopy supported by molecular simulations. We observe solute–solvent interaction weakening in the same order: methanol < acetonitrile < ethyl acetate, quantifying the weaker solvent–solute interactions that permit the formation of larger prenucleation clusters. Our results support the hypothesis that nucleation is easier in weaker solvents because weak solute–solvent interactions favor growth of large clusters, as opposed to relying solely on ease of desolvation.

## Abstract 2

Molecular clustering of salicylamide in ethyl acetate, methanol, and acetonitrile was investigated using photon correlation spectroscopy. To understand solvent−solute effects, IR spectroscopy was performed supported by molecular simulations. Nucleation gets easier in weaker solvents because of weak solute−solvent interactions which favor growth of large clusters.

## 1 Introduction

Crystallization is used for the separation and purification of a variety of inorganic and organic compounds. The fundamentals of crystal formation include nucleation, the initial formation of the smallest crystalline entity, followed by crystal growth, progressing to the desired product particle size. Crystal nucleation has a significant influence on the product crystal size distribution and the crystalline structure of the solid product. For many decades, classical nucleation theory (CNT)^1−6^ has been used to describe crystal nucleation in solution. CNT assumes that crystal nucleation occurs when prenucleation clusters reach the size of the so-called “critical nucleus”. The critical nucleus is the smallest crystal that is thermodynamically stable in the host supersaturated solution. The theory assumes that prenucleation clusters have crystalline structure and are formed by random molecular fluctuations in the solution. However, there are several observations related to crystal nucleation that are not well described by CNT, and consequently the theory has been challenged by so-called “non-classical” crystallization mechanisms. According to the two-step nucleation theory,^7^ molecular assemblies of the order of nanometers to a few micrometers are formed in undersaturated, saturated, and supersaturated solutions. The two-step theory suggests that these assemblies are solute-rich but not necessarily free of solvent molecules and that they are disordered, liquid- or gel-like materials. Upon nucleation, a molecular rearrangement takes place in these assemblies leading to an increase in order parameters^8^ and the formation of nuclei.

Various studies have shown that nanometer-sized clusters with liquid-like properties can be found in concentrated solutions of large organic molecules, e.g., proteins, and of small molecules.^7,9−12^ Photon correlation spectroscopy (PCS) studies have shown that these clusters can be characterized as near-spherical, discrete domains that present higher solute density in comparison to the rest of the solution, containing 10^3^ to 10^8^ solute molecules.^13−15^ These solute-rich molecular assemblies are well dispersed within the bulk solution and should not be considered a separate phase.^16^ Previous works reported broad size distributions of clusters, with ranges of several hundred nanometers, in undersaturated aqueous solutions of common small organic molecules, such as various amines and amino acids,^17,18^ citric acid, urea, and glucose.^13−15^ Mesoscale domains were found in undersaturated and supersaturated aqueous solutions with radii ranging from 50 to 300 nm for two amino acids, glycine and dl-alanine.^17^ Several studies have given different names to molecular assemblies that precede crystal nucleation, such as nanoclusters, mesoclusters, or prenucleation clusters. We use the latter term here. Other than PCS, methods such as nanoparticle tracking analysis (NTA) are powerful for direct determination of the size of mesoscale clusters in the prenucleation stage of organic compounds through their solvodynamic diameter just like the PCS.^19,20^ The internal structures of the prenucleation clusters have also been studied by small-angle X-ray scattering (SAXS)^21^ or small-angle neutron scattering (SANS)^22^ with increasing solute concentration. On the other hand, transmission electron microscopy (TEM) offers real-time tracking of prenucleation clusters by visualization; methods such as cryo-TEM have proven extremely useful in delineating nucleation pathway.^23,24^

Recent PCS measurements of fenoxycarb and salicylic acid in organic solvents^25^ tracked the cluster size as a function of time in solutions at different concentrations, including supersaturated conditions. Nanometer-sized clusters were found from the very beginning in all solutions at all solute concentrations. The cluster size increased systematically with increasing concentration and with increasing time over days. The cluster size at equal mole fraction *x* of each solute in the different solvents increased with decreasing solubility. In the saturated solution, the cluster size for each solute did not show a clear dependence on the solvent, but salicylic acid clusters were smaller than those of fenoxycarb. It was found that the cluster size in the supersaturated solution (at relative solute concentration or degree of solution saturation *x*/x* = 1.05, where x* is the solubility) correlates with the nucleation behavior of each compound in the different solvents, i.e., the larger the clusters, the easier the nucleation. Across the two solutes and the different solvents, the cluster size at equal supersaturation (*x*/x* = 1.05) decreased proportionally with increasing interfacial energy, as stronger solute–solvent interaction makes nucleation more difficult. These results indicate that the ease of nucleation is not primarily related to the ease of desolvation but is rather because strong solvation leads to smaller prenucleation clusters, so nucleation is more difficult, and we obtain smaller clusters in those solvents.

**Figure 1:**
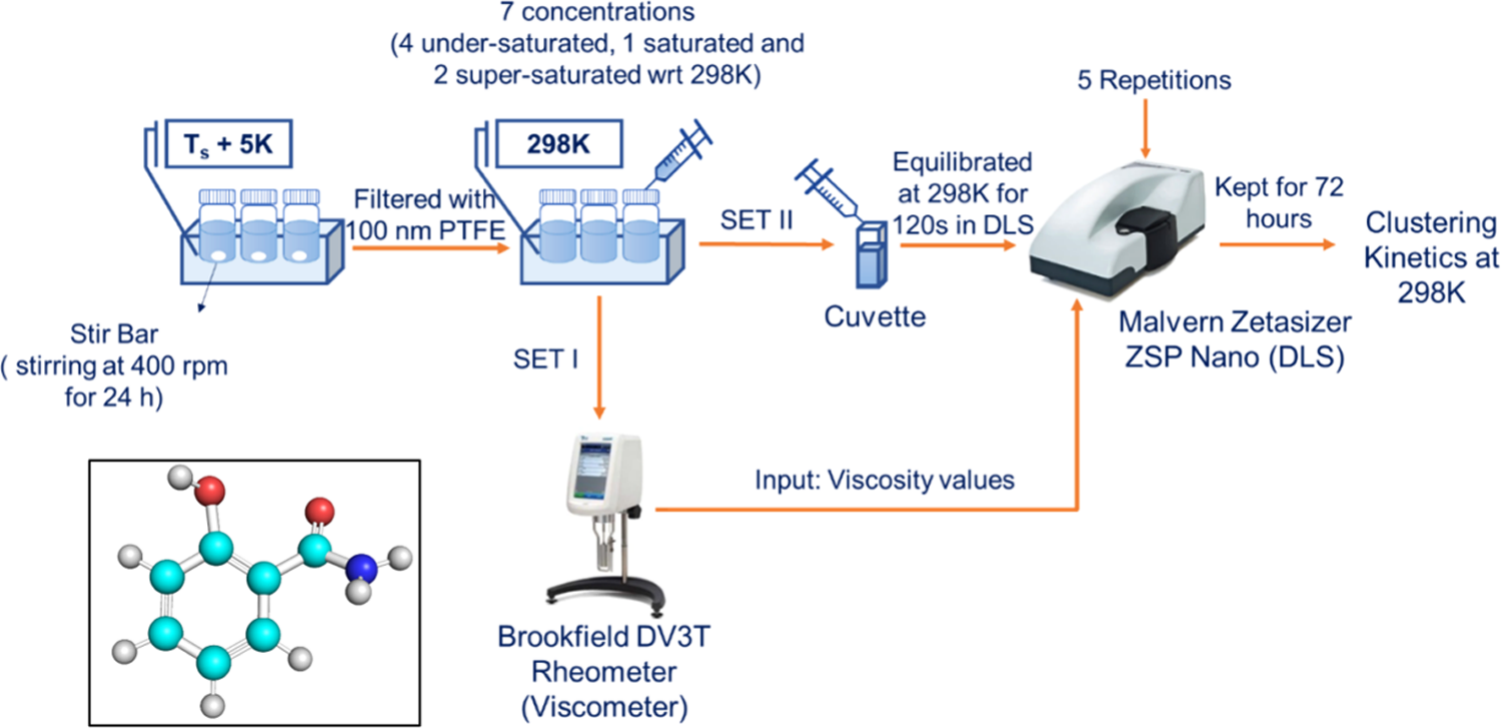
Lab protocol developed for the clustering experiments. The molecular structure of salicylamide is given in the inset in ball and stick representation. Carbon atoms are colored cyan, oxygen atoms are red, nitrogen is blue, and hydrogen atoms are colored gray.

In the present work, molecular clustering of salicylamide in three different organic solvents is investigated and compared to the corresponding nucleation behavior. The salicylamide molecule (2-hydroxybenzamide, shown in Figure 1) has analgesic, anti-inflammatory, and antipyretic properties. Sasada et al.^26^ determined the crystal structure of the monoclinic form of salicylamide including a polymorph formed at high pressure.^27^ Recently, several groups have reported on the solubility,^28^ nucleation,^29,30^ and growth^31^ of salicylamide in different solvents, but the early-stage prenucleation clustering has not been examined to date. In the present study, the clustering of salicylamide in three different organic solvents, ethyl acetate, methanol, and acetonitrile, at 298 K is investigated by PCS. We have examined the influence of time and concentration on salicylamide nucleation. Additionally, to understand the influence of solute–solvent interactions of salicylamide in the three solvents, we characterized the solutions by infrared (IR) vibrational spectroscopy and used molecular dynamics (MD) simulations to resolve the atomic-scale mechanism of salicylamide cluster formation in saturated solutions. To the best of our knowledge, the solute–solvent interactions of salicylamide in different solvents have not been reported in previous studies. Thus, in this work, we map the full relationship between nucleation behavior, cluster size, and the molecular solute–solvent interactions.

## 2 Experimental Methods

### 2.1 Materials

Salicylamide (CAS Number 65–45–2) with 99% purity was used for all experiments. Methanol was used with 99.9% purity, ethyl acetate with 99.7% purity, and acetonitrile with 99.9% purity. The solute and solvents were purchased from Sigma-Aldrich and were used as received. The experimental protocol for clustering of salicylamide is shown in Figure 1.

### 2.2 Preparation of Solutions

**Figure 2:**
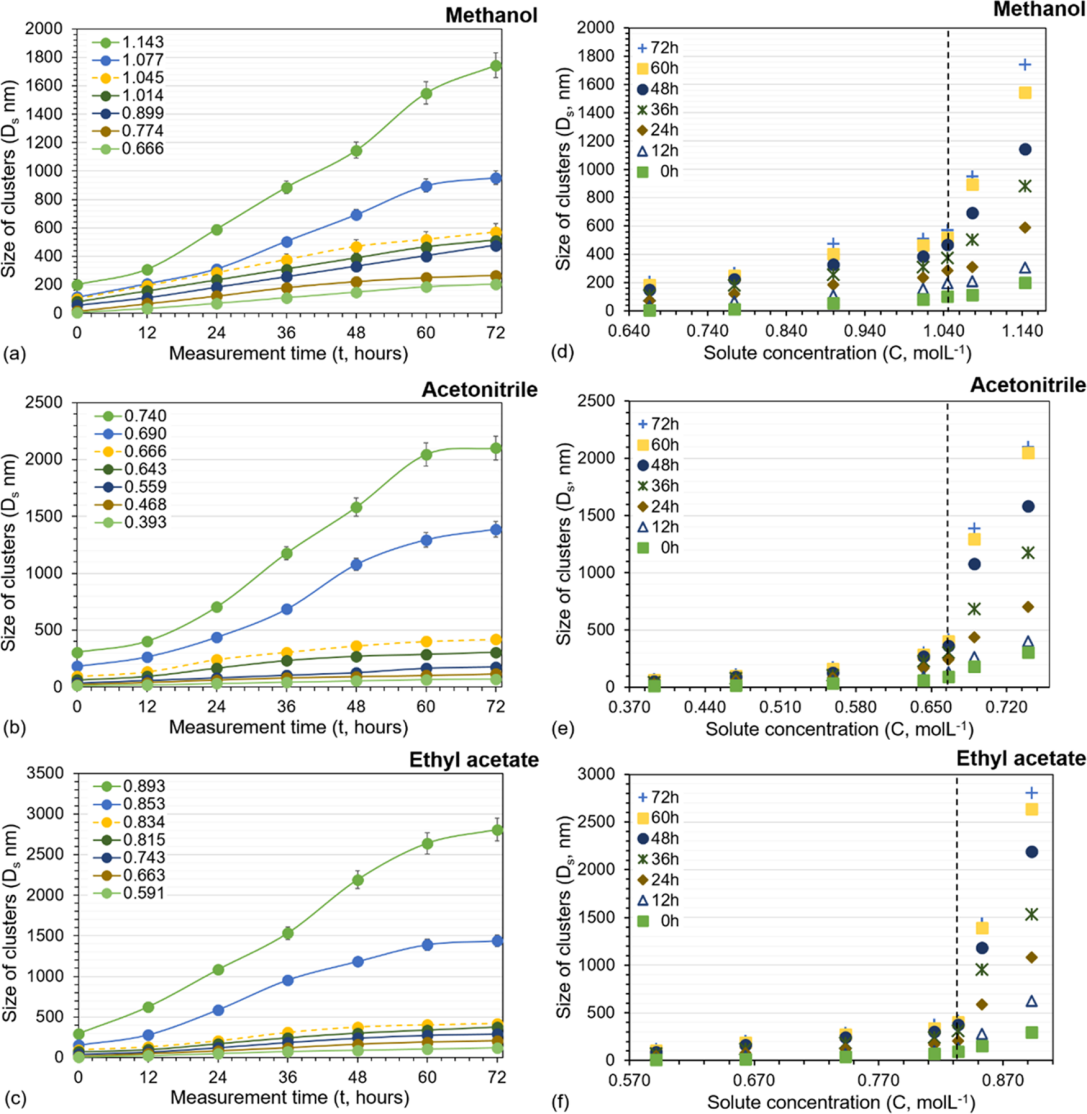
Mean solvodynamic diameter of salicylamide nanoclusters vs measurement time (a–c) and vs solute concentration (*x*, mol/L) (d–f) in methanol, acetonitrile, and ethyl acetate solutions. Error bars show standard deviations across the five measurements. Dashed lines mark saturated solution: (left-hand-side diagrams as trend lines; right-hand-side diagrams as vertical lines) of salicylamide acid at 298 K in that solvent.

100 mL stock solutions (100 mL) of salicylamide in the three different solvents were prepared in 250 mL glass bottles with seven different concentrations: in acetonitrile from 0.393 to 0.740 mol/L, in methanol from 0.666 to 1.143 mol/L, and in ethyl acetate from 0.591 to 0.893 mol/L. Before use, the solvents were filtered with heated syringes and 0.2 μm PTFE filters from VWR. Based on solubility data for salicylamide,^28^ the stock solutions were then kept at 5 °C above their saturation temperature for 24 h under 400 rpm stirring to ensure complete dissolution. After 24 h, different volumes (10 and 2 × 25 mL) of the solutions were extracted and filtered with a preheated 0.1 μm PTFE filter and syringed into three clean and heated 30 mL vials. The contents of one vial (10 mL) were used for viscosity measurements and the contents of the other two (25 mL) for PCS solvodynamic diameter measurements at temperature 298 K. For PCS measurements, a sample of solution from 25 mL vials was extracted using a 1 mL glass syringe and filtered through 0.1 μm PTFE filters into cuvettes for measurements every 3 h over a period of 72 h. These samples were maintained at 298 K without stirring in a second water bath. Of the seven different concentrations, four solutions were undersaturated, one was saturated and two were supersaturated at the temperature of the clustering measurements, i.e., 298 K. The exact concentration values in each solvent are apparent from Figure 2. For stirring, Sigma-Aldrich magnetic stir bars of polygon shape measuring 1/2 × 1/8 in. were used. The temperature was kept constant in all of the solutions using water baths with a C2C cooling unit and submerged magnetic plates from Grant. Procedures for the cluster size determinations using Malvern Zetasizer ZSP Nano instrument and solution viscosity measurements using Brookfield DV3TRVTJ Rheometer can be found in Notes S1 and S2.

### 2.3 Infrared Spectroscopy Investigations

Infrared (IR) spectroscopy was performed on saturated salicylamide solutions prepared based on the solubility at 298 K. The solution spectra were collected using a Mettler Toledo *in situ* probe IR fitted with a silver halide probe composed of diamond composite and mercury cadmium telluride (MCT) detector cooled with liquid nitrogen. For each spectrum, 250 scans were collected from 2000 to 650 cm^–1^ at 4 cm^–1^ resolution using iC IR software, version 4.3. Background solvent spectra were subtracted. All of the spectral data were collected at 298 K. The spectra of salicylamide solid material were measured using a PerkinElmer100 spectrometer, equipped with a universal attenuated total reflectance (ATR) accessory (single reflection and diamond/zinc selenide material) and a lithium tantalate detector. 256 scans were collected in the spectral region of 4000–400 cm^–1^ with a resolution of 4 cm^–1^.

### 2.4 Atomistic Molecular Dynamics Simulations

The salicylamide molecule was represented by force field partial charges and parameters obtained from the CHARMM General Force Field (CGenFF).^32^ We derived the interatomic potentials from the crystal polymorph (see Figure S3a; monoclinic, space group: *P*2(1)/*c*; CCDC^33^ code 1545142^34^) using the solid-state “General Utility Lattice Program” (GULP^35^) program and optimized the Lennard-Jones (LJ) parameters to be compatible with CHARMM36m^36^ force field (see Note S3 for details).

A total of 1.5 μs of room-temperature molecular dynamics (MD) was performed to model salicylamide clustering during 500 ns of equilibrated and unconstrained constant pressure room-temperature dynamics in the three saturated solutions. In the first simulation cell, 125 salicylamide molecules were placed in a solvent box of initial volume 201.5 nm^3^ containing 3000 methanol molecules at their experimental density of 0.645 g/mL, matching the experimental solute:solvent ratio of 1:24 (referring to *x* = 1.045 mol/L). The second cell of volume 689.3 nm^3^ contained 4250 ethyl acetate molecules (ρ = 0.833 g/mL) to give a molecular ratio of 1:34 (referring to *x* = 0.666 mol/L). The third cell of volume 401.04 nm^3^ contained 4625 acetonitrile molecules (0.748 g/mL) yielding a ratio of 1:37 (referring to *x* = 0.834 mol/L). The minimum distance between any salicylamide atom and any box edge was kept at 15 Å. Solvent boxes of methanol, ethyl acetate, and acetonitrile were pre-equilibrated at 1 bar for 5 ns and then used to solvate salicylamide. MD simulations were performed using the Gromacs 2018.4^37,38^ code with an integration time step of 2 fs implemented in the velocity Verlet integrator^39^ with bond lengths to hydrogen constrained using the LINCS^40^ algorithm for salicylamide and SETTLE^41^ for solvent molecules. Snapshots were saved every 20 ps. Long-range electrostatics were treated by the Particle mesh Ewald (PME) method.^42^ Salicylamide and solvents were coupled separately to an external heat bath (298 K) with a coupling time constant of 1 ps using the velocity rescaling method.^43^ All systems were energy-minimized and equilibrated for 1 ns in constant-volume NVT ensemble followed by another 1 ns of NPT equilibration with the reference pressure at 1 bar and a time constant of 4 ps using the Berendsen barostat.^44^ The 500 ns production runs were carried out in constant pressure NPT ensemble using the Parrinello–Rahman barostat.^45^

### 2.5 Solvation Free Energy Calculations

The solvation free energy is the free energy change associated with moving a single molecule from a gas-phase environment into the solution. We employ two methods for solvation free energy calculations. The alchemical free energy method^46^ uses a thermodynamic integration (TI) model (see Figure S1) in explicit solvent while the Poisson–Boltzmann free energy method uses an implicit continuum solvation model,^47,48^ as detailed in Notes S4 and S5.

## 3 Results

### 3.1 Clustering of Salicylamide in Different Solvents

Figure 2 shows the cluster sizes obtained for salicylamide in different solvents against the measurement time (in hours) and at different solute concentrations (*x* in mol/L). Irrespective of the solvent and at all concentrations, the size of salicylamide clusters increases monotonically with time over the three-day period. In most cases, the slope of the curve plateaus at a maximum size within ca. 60–72 h. In all three solvents and at every stage, the cluster size of salicylamide increases with solute concentration from undersaturated solutions to saturated and supersaturated solutions. In all three solvents, the slope of the concentration dependence increases with concentration, especially when moving close to and above the saturation concentration. There was no cloudiness observed in the supersaturated solutions in methanol and acetonitrile within the experimental time frame of 72 h, and some small cloudiness was observed in ethyl acetate solutions at higher supersaturations.

**Figure 3:**
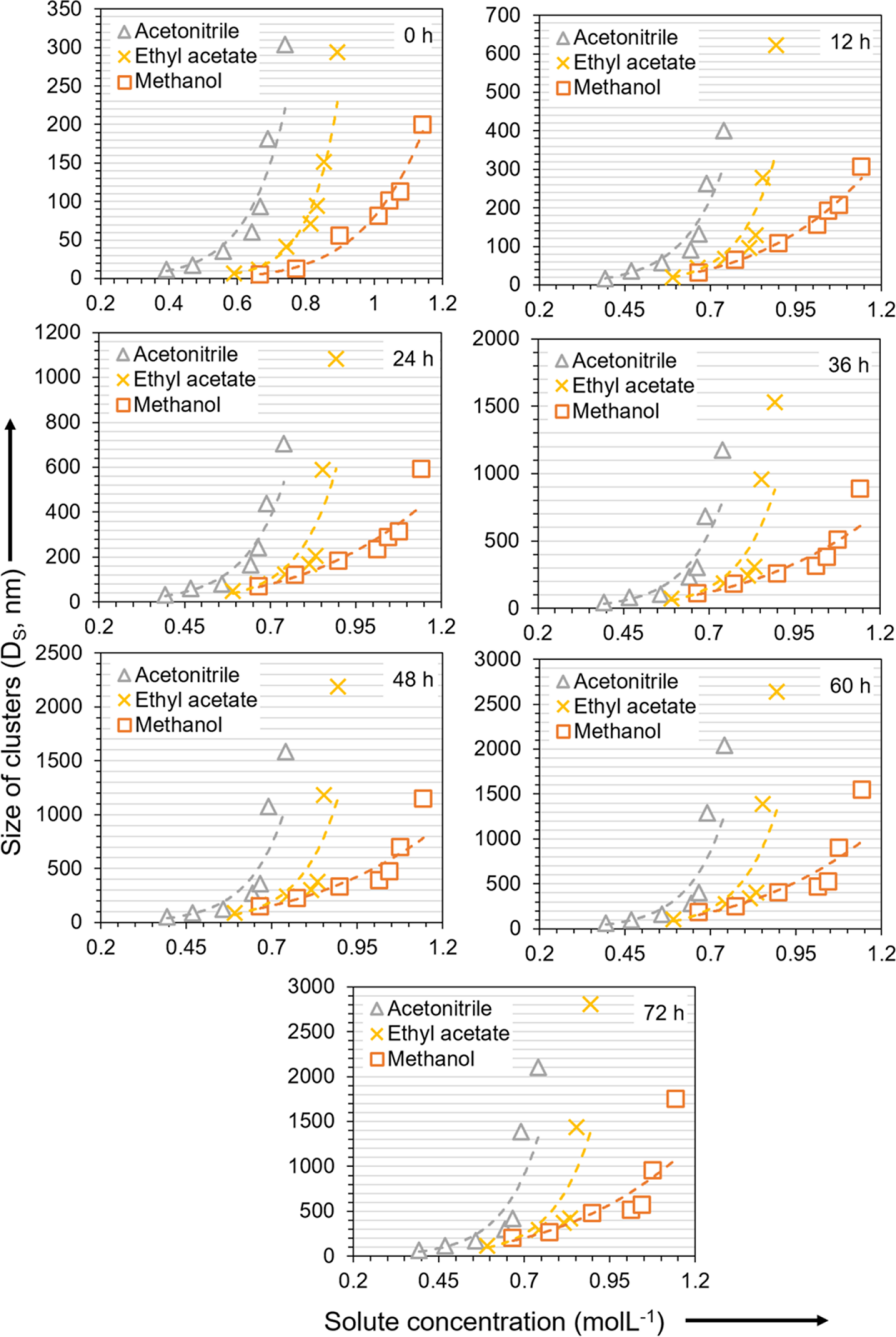
Cluster size vs solute concentration (*x*) at different times in the three solvents. Methanol (orange, orange box), acetonitrile (gray, gray delta), and ethyl acetate (yellow, yellow times).

In Figure 3, the sizes of salicylamide clusters obtained in the three solvents at different solute concentrations (*x*) are compared at different times. Regardless of time, the cluster size at equal solute concentration increases in the order: methanol < ethyl acetate < acetonitrile. Notably, the solubility of salicylamide in the three solvents decreases in the same order.

**Figure 4:**
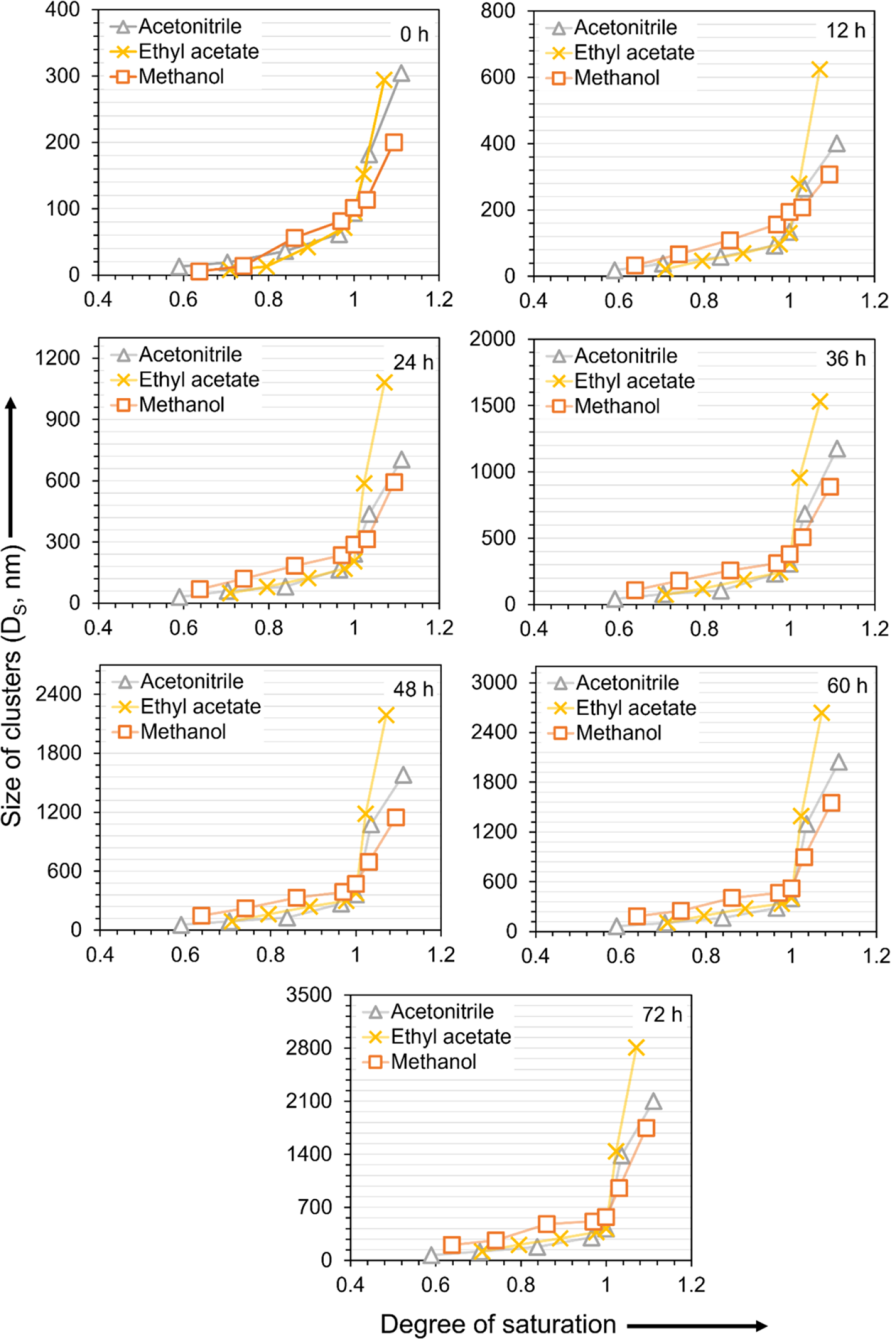
Cluster size vs degree of saturation, i.e., relative solute concentration (*x*/x*) at different times. Methanol (orange, orange box), acetonitrile (gray, gray delta), and ethyl acetate (yellow, yellow times).

In Figure 4, the concentration scale used in Figure 2 is normalized against the solubility (x*) and accordingly shows the relative solute concentration or degree of solution saturation (*x*/x*). The dependence on the solvent is much weaker when compared at equal relative concentration, especially at concentrations below saturation. Relative concentration *x*/x* < 1 means the solution is in undersaturated state and relative concentration *x*/x* > 1 means the solution is in supersaturated state. At equal relative concentration below saturation, i.e., *x*/x* < 1, the clusters tend to be the largest in methanol and the smallest in acetonitrile, with some deviations at very short times. At supersaturated conditions, the order with respect to the solvent at equal relative concentration tends to be reversed, with the clusters being the largest in ethyl acetate and the smallest in methanol. This reverse pattern of clustering is consistent with previous observations for fenoxycarb and salicylic acid.^25^ From Figure 4, the cluster size in the saturated solutions appears to be relatively independent of the solvent.

**Figure 5:**
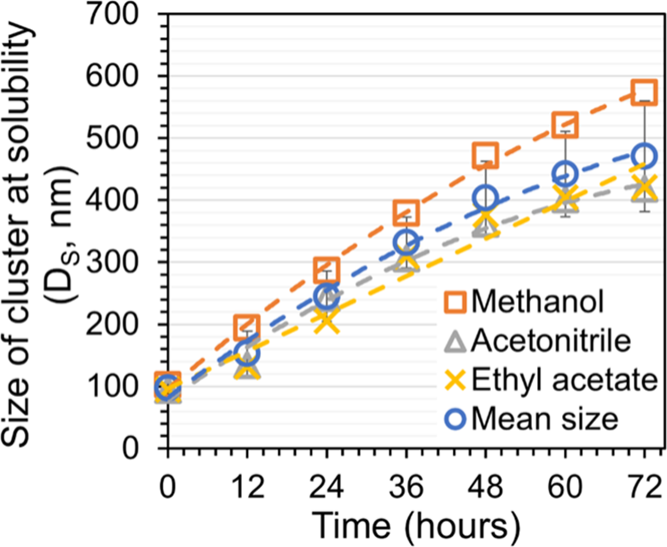
Cluster size in a saturated solution of salicylamide in the three solvents. Lines are drawn to guide the eye. Methanol (orange, orange box), acetonitrile (gray, gray delta), and ethyl acetate (yellow, yellow times). Mean size of salicylamide clusters (blue, blue circle open) with variation bars in black.

In a saturated solution of all solvents, the cluster size increases with time, as further detailed in Figure 5. The clusters in ethyl acetate and acetonitrile are somewhat smaller than in methanol, but overall, the cluster size is relatively independent of the solvent. The average size of salicylamide clusters in the saturated solution of the three solvents is ∼400 ± 60 nm after 48 h.

### 3.2 Spectroscopic Monitoring of Solute–Solvent Interactions

**Figure 6:**
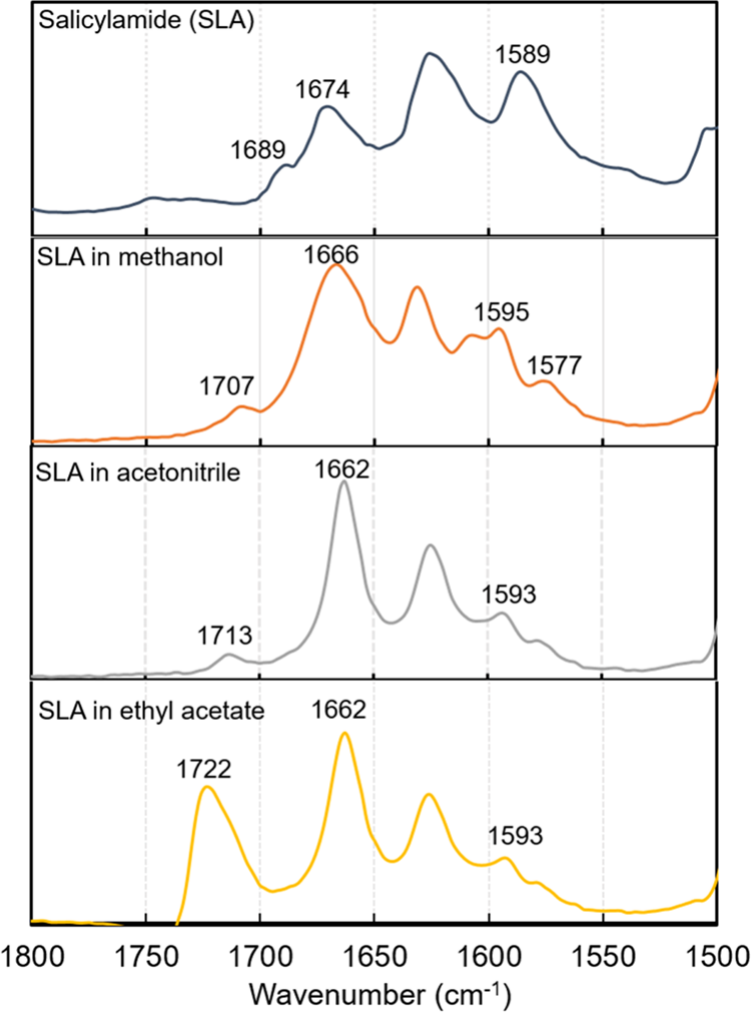
IR spectra of pure solid salicylamide (blue) and of salicylamide in saturated solution in methanol (orange), acetonitrile (gray), and ethyl acetate (yellow) (top to bottom).

The Fourier transform infrared (FTIR) spectra of pure solid salicylamide and of salicylamide solutions saturated at 298 K^28^ in the three solvents methanol, ethyl acetate, and acetonitrile are shown in Figure 6. For the pure solid, we observe stretching peaks at 1674 cm^–1^ (amide I) and at 1589 cm^–1^ (amide II),^49^ and a smaller peak at 1689 cm^–1^. Even though they are slightly shifted from the pure solid, the first two peaks are within 1–4 cm^–1^ of each other in the three solutions. A more significant influence of the solvent is seen only for the peak at the highest wavenumber, i.e., the peak at 1689 cm^–1^ in the solid spectra. In the crystal structure, the main building block is centrosymmetric dimers formed by hydrogen bonding to the carbonyl group (Figure 1). This restricts the carbonyl stretching vibration represented by the main amide I peak, and the intramolecular bonding to the alcohol group further reduces the wavenumber. The smaller peak at the higher wavenumber of 1689 cm^–1^ is interpreted as a certain fraction of the carbonyl groups being less tightly constrained, perhaps not fully dimerized in the solid, or not engaged in intramolecular hydrogen bonding. In all three solvents, this peak is clearly shifted to higher wavenumbers. This carbonyl peak shifts to a higher wavenumber in the order: methanol (1707 cm^–1^) < acetonitrile (1713 cm^–1^) < ethyl acetate (1722 cm^–1^), reflecting corresponding reduced interactions with the solvent in the same order as stronger bonds with reduced solvent screening absorb at higher wavenumbers.

### 3.3 Estimation of Solute–Solvent Interactions from Solvation Free Energies

To further investigate the trend obtained from FTIR in Section 3.2, the solvation free energies of a single molecule of salicylamide in different solvents were estimated from the MD structures using two different methods. The results obtained using the Poisson–Boltzmann (PB) continuum solvation method and alternative alchemical free energy calculations are summarized in Tables 1 and 2, respectively. See Note S5 for details of the simulations. From both the alchemical and PB methods, the magnitude of the solvation free energy values for salicylamide in different solvents was found to decrease in the order: methanol > acetonitrile > ethyl acetate, indicating that the strength of solute–solvent interactions for salicylamide in different solvents decreases in the same order. For further comparisons, the values of *G*_solv2_ are used as the lower magnitudes are more in line with experimental values.^28^ We also performed MD simulations of randomly dispersed 125 salicylamide molecules (experimental saturated conditions) to mimic experimental cluster formation in the three solvents (see Section 2.4) and estimated the solvation free energies during clustering of salicylamide (discussed below in Section 3.4).

**Table 1:** Solvation Free Energy for Salicylamide in Different Solvents Calculated Using the Alchemical Free Energy Method (See Note S4 for Details of the Calculation)

solvent	ideal (kJ/mol)	van der Waals (kJ/mol)	electrostatic (kJ/mol)	solvation *G*_solv1_ (kJ/mol)
methanol	186.21	–24.48	–212.02	–50.258
acetonitrile	190.52	–23.42	–216.44	–49.308
ethyl acetate	190.45	–29.19	–207.06	–45.769

**Table 2:** Solvation Free Energy for Salicylamide in Different Solvents Calculated Using the Poisson–Boltzmann Continuum Solvation Method (See Note S5 for Details)

solvent	van der Waals (kJ/mol)	electrostatic (kJ/mol)	solvation *G*_Solv2_ (kJ/mol)
methanol	159.68 ± 15.14	–426.69 ± 18.14	–22.11 ± 2.33
acetonitrile	118.21 ± 18.93	–433.05 ± 14.11	–21.36 ± 2.21
ethyl acetate	162.45 ± 13.03	–432.65 ± 13.32	–13.33 ± 1.24

### 3.4 Predictive Modeling of Clustering Behavior in Different Solvents at Saturated Salicylamide Concentration

We further examined the MD trajectories (see Section 2.4, Note S3, and Figures S2 and S3 for details of these simulations) to probe the balance of solute–solute and solute–solvent interactions in directing the size of molecular clusters formed at saturated concentrations of salicylamide.

**Figure 7:**
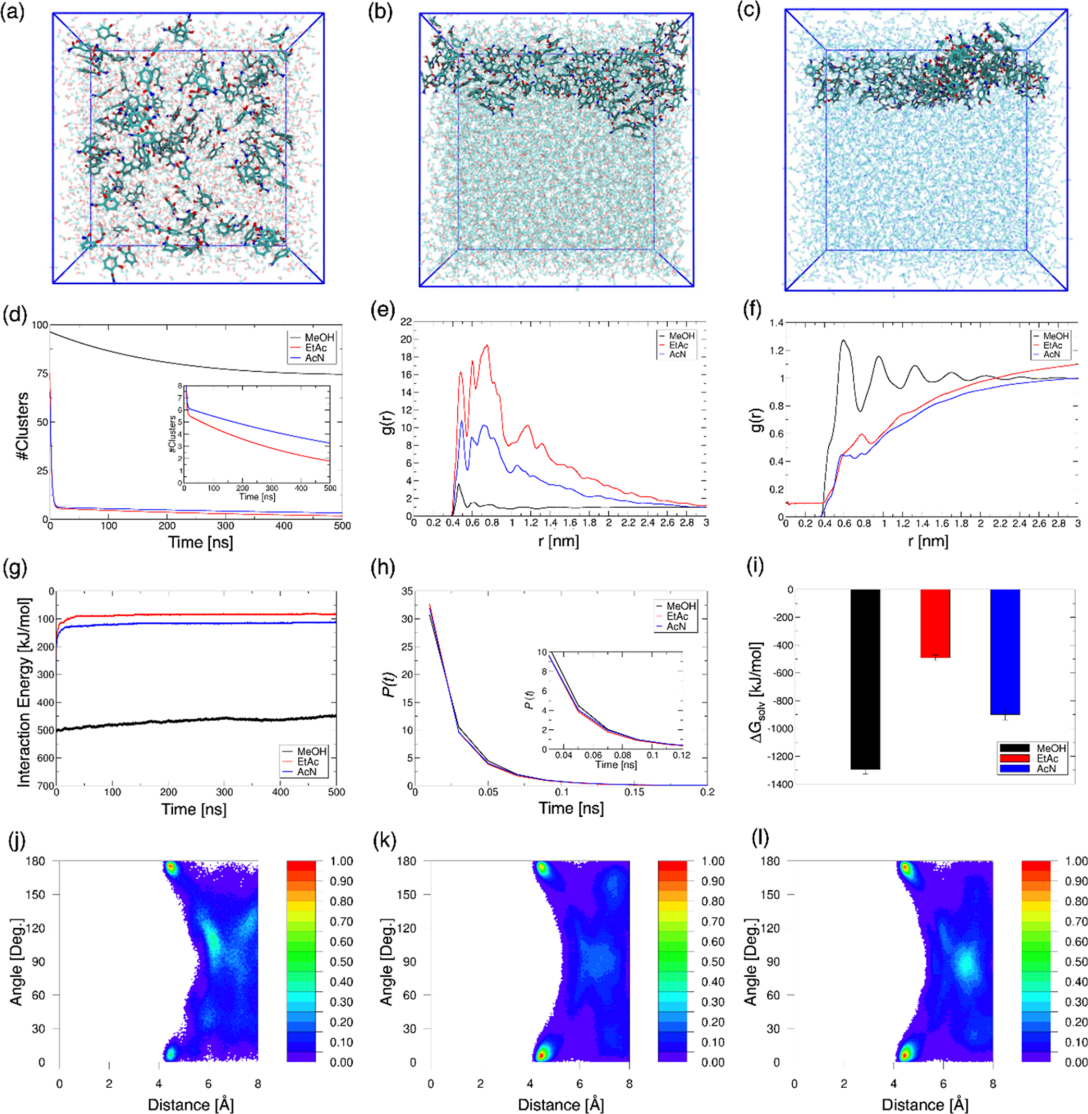
(a–c) Final structures after 500 ns of free dynamics of 125 salicylamide molecules in three different solvents: (a) methanol (MeOH), (b) ethyl acetate (EtAc), and (c) acetonitrile (AcN). (d) Number of salicylamide clusters formed as a function of time in the three solvents. A second-order fit to the data is shown, with the raw data given in Figure S4a. The inset is a zoom-in on the cluster formation in EtAc and acetonitrile AcN data, highlighting the distinct kinetic and thermodynamic behaviors of these assemblies. A minimum cutoff distance of 3.5 Å between salicylamide molecules was considered for cluster formation. (e, f) Radial distribution functions (*g*(*r*)) showing structuring of (e) salicylamide–salicylamide and (f) salicylamide–solvent contacts in the three solvents. (g) Salicylamide–solvent interaction energies (electrostatic + vdW) in three solvents. The data are normalized per molecule of salicylamide. (h) Comparison of the lifetime (*P*(*t*)) of salicylamide–salicylamide H-bonds formed in three solvents at a donor–acceptor cutoff distance of 3.5 Å and a hydrogen-donor–acceptor angle of 30°. The inset shows a close-up of the H-bond lifetimes against time. (i) Solvation free energy (*G*_solv_) of the salicylamide clusters. The mean and standard error of the mean (SEM) are calculated over the last 100 ns of dynamics. (j–l) Computed free energy landscape for salicylamide assembly in (j) MeOH, (k) EtAc, and (l) AcN mapped over the first 10 ns of dynamics, highlighting the creation of π–π stacks.

The MD simulations reveal that salicylamide forms superstructures in EtAc and AcN (Figure 7b,c) while salicylamide forms smaller clusters in MeOH (Figure 7a). We observe that 2–4 nanoclusters are immediately formed in EtAc and AcN within the first 10 ns of dynamics (Figure 7d and see also Figure S4a) with EtAc producing slightly larger clusters than AcN (see inset in Figures 7d, and S4b). Our data indicates weaker solvation of salicylamide in EtAc, as predicted from the solvation free energies of a single molecule of salicylamide (see Tables 1 and 2) and measured from the experimentally observed rank ordering of size of salicylamide prenucleation clusters in the different solvents (Figure 7i).

The MD simulations provide a map of superstructure formation in the different solvents. The radial distribution function *g*(*r*) or pair correlation function was extracted by considering a maximum distance (*r*_max_) of 3 nm separation between the center of mass (COM) of two salicylamide molecules and salicylamide and solvent molecules. The *g*(*r*) maps show the maximum probable number density (of molecular contacts) peak at 0.45 nm distance of separation between salicylamide molecules in methanol, and 0.5 nm distance in ethyl acetate and acetonitrile (Figure 7e) with maximum peak height in ethyl acetate. This is followed by minimal structuring with poor peak resolution in MeOH at a larger distance of separation between salicylamide molecules, but in EtAc and AcN, we see large peaks at 0.7 and 1.1 nm, highlighting the salicylamide superstructure formation in these solvents. The *g*(*r*) for solute–solvent distribution (Figure 7f) shows a maximum peak height (with 0.6 nm distance of separation) in MeOH, quantifying the salicylamide–MeOH ordering that stabilizes small clusters. Further, the plateau in MeOH at longer solute–solvent interaction distances shows that their interactions are uncorrelated, indicating that salicylamide can take up these inclusion spaces at experimental time scales.

Model-predicted salicylamide–solvent interaction energies (Figure 7g) show the intermolecular contacts that direct the growth of small vs larger clusters of salicylamide, with least favorable interactions predicted with EtAc followed by AcN and most favorable salicylamide interactions with MeOH. At the same saturated concentration, the lifetime (*P*(*t*)) of salicylamide–salicylamide H-bonds existence is seen to be more slowly decaying in MeOH than in EtAc and in AcN (see the inset in Figure 7h). On the other hand, the lifetime of salicylamide–solvent H-bonds existence (Figure S4c) is predicted to be longer in MeOH than in other solvents, which points toward an intricate balance between solute–solute and solute–solvent H-bonds in directing the growth of the salicylamide clusters.

Finally, we investigated the contribution of π–π stacking of the salicylamide benzene rings to the superstructure formation. The free energy maps of the angle vs distance of salicylamide predict a relatively sparse landscape in methanol for the full 500 ns of dynamics (Figures S4d–f). To further probe this, we investigated the first 10 ns dynamics of salicylamide π–π interactions, where the clusters start forming in EtAc and AcN (Figure 7j–l). We observe prominent parallel π–π stacking (at close to 0 or 180° and <4.5 Å distance) of salicylamide in both EtAc (Figure 7k) and AcN (Figure 7l), which is less evident in MeOH (Figure 7j), with some degree of T-shaped stacking also in both MeOH and AcN, but less in EtAc. Taken together, our models of salicylamide clustering in saturated solutions help explain the trend seen in experiments (see Figure 4) with the propensity for salicylamide superstructure formation following the order: ethyl acetate > acetonitrile > methanol, owing to the free energy balance between solute–solute and solvent–solute interactions. Formation of smaller short-lived clusters of salicylamide observed in MeOH could be attributed mainly to the sparse population of salicylamide–salicylamide H-bonds within the loose, methanol-rich network predominated by salicylamide–MeOH H-bonds, with little contribution from π–π interactions. By contrast, the larger, long-lived clusters in EtAc and AcN can be attributed mainly to π–π stacking as solvent is expelled due to weaker solute–solvent interactions.

## 4 Discussion

### 4.1 Clustering Behavior in Different Solvents

We observe that the clustering of salicylamide in the three organic solvents follows the trends observed previously for fenoxycarb and salicylic acid.^25^ Respective of the solvent, the size of salicylamide clusters increases with solute concentration. At concentrations above saturation, the increase is much faster than that at concentrations below saturation, as most clearly observed for ethyl acetate solutions. The sizes obtained in this study in undersaturated, saturated, and supersaturated solutions are consistent with what has been observed previously for fenoxycarb and salicylic acid.^50,51^ Furthermore, in all solvents and at all concentrations, the rate of cluster size growth levels off at increasing time.

The influence of the solvent on the cluster size is significant when compared with equal solute concentration, with the cluster size increasing in the same order as the solubility decreases. However, when the concentration is normalized by the solubility, the influence of the solvent is much weaker. This suggests that solute concentration in relation to the solubility, i.e., degree of solution saturation, (*x*/x*), provides a more sensitive measure than the molecular concentration. Specifically, it better reflects the molecular-level conditions relevant to prenucleation clustering in different solvents. As observed for fenoxycarb and salicylic acid,^25^ the order of the solvents with respect to cluster size at equal relative concentration below saturation is reversed when pushed above saturation. In supersaturated solutions, the cluster size at equal relative concentration increases in the order: methanol < acetonitrile < ethyl acetate. In the saturated solution, the cluster size is only weakly dependent on the solvent and is approximately 400 nm after 48 h. The size is approximately the same as the corresponding value obtained for salicylic acid but is clearly lower than the 900 nm cluster size found for fenoxycarb^25^ which reflects the corresponding ratio of molecular weights.

**Figure 8:**
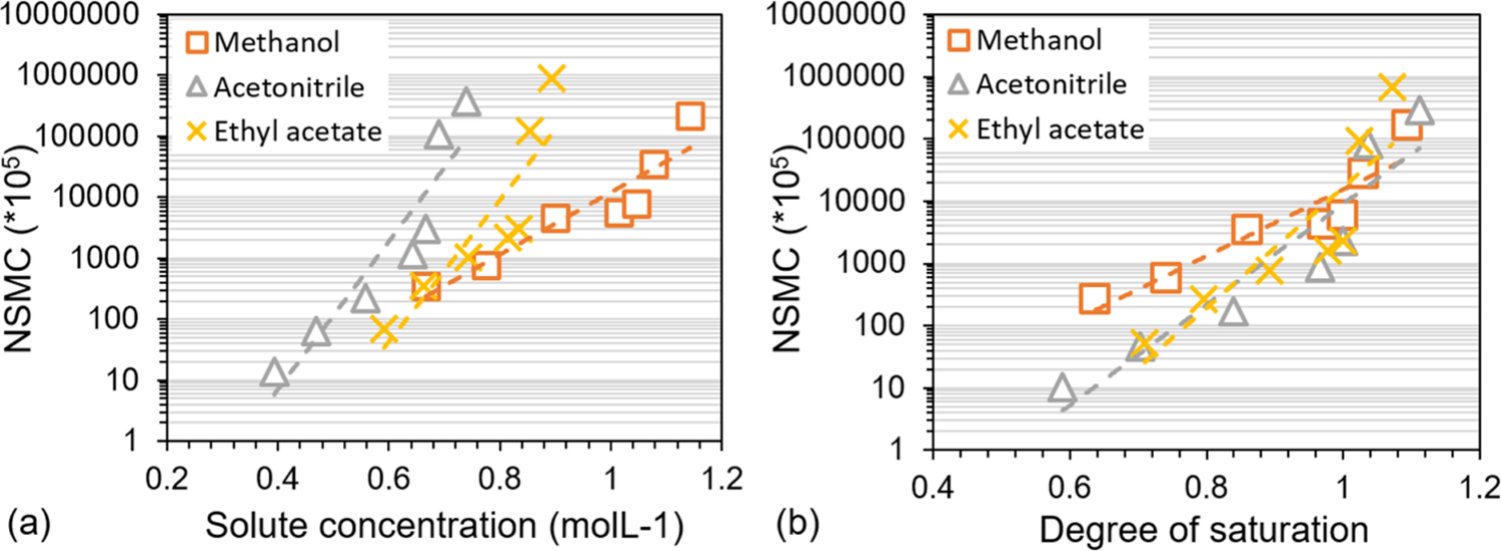
NSMC calculated using eq 1 obtained after 72 h vs solute concentration and degree of saturation. Trend lines shown are exponential functions. Methanol (orange, orange box), acetonitrile (gray, gray delta), and ethyl acetate (yellow, yellow times).

The number of solute molecules per cluster, assuming the clusters to be solute-rich^52^ spherically shaped entities having a diameter equal to the solvodynamic diameter, can be estimated as
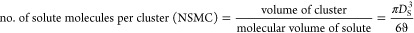
1where ϑ is the molecular volume of the solute. For all calculations of NSMC, the molecular volume of salicylamide was taken as 0.169 nm^3^ obtained using Mercury software v.4.0.0 and crystallographic information file (cif) SALMID01. The NSMC obtained after 72 h for different concentrations is shown in Figure 8a and for different relative concentrations, i.e., normalized by solubility in Figure 8b. At saturation, the clusters contain on the order of 10^3^ to 10^4^ molecules, approximately the same as found for fenoxycarb and salicylic acid.^25^

The rate of the cluster size increase in terms of NSMC can be estimated by a simple difference calculation (eq 2)
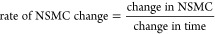
2If the rate of increase of the cluster size is governed by simple diffusional mass transfer from the solution to the cluster surface, then the frequency of attachment of molecules is proportional to the area of these clusters. In that case, the rate of increase of the NSMC [molecules/*h*] equals the rate of mass flux (DC/*r*) (assuming no convection) multiplied by the cluster surface area (4π*r*^2^) and the Avogadro number, and thus becomes directly proportional to the cluster radius. Accordingly, if the cluster size increase is controlled by molecular diffusion, the rate of the NSMC increase divided by the cluster radius should be constant over time. The corresponding results for different concentrations in the three solvents (Figure S5) show that the ordinate increases quite substantially with time for each condition, even though the increase gradually levels off at longer times. Hence, the results do not support the idea that the cluster size increase is governed by ordinary molecular diffusion from the liquid to the cluster surface.

**Figure 9:**
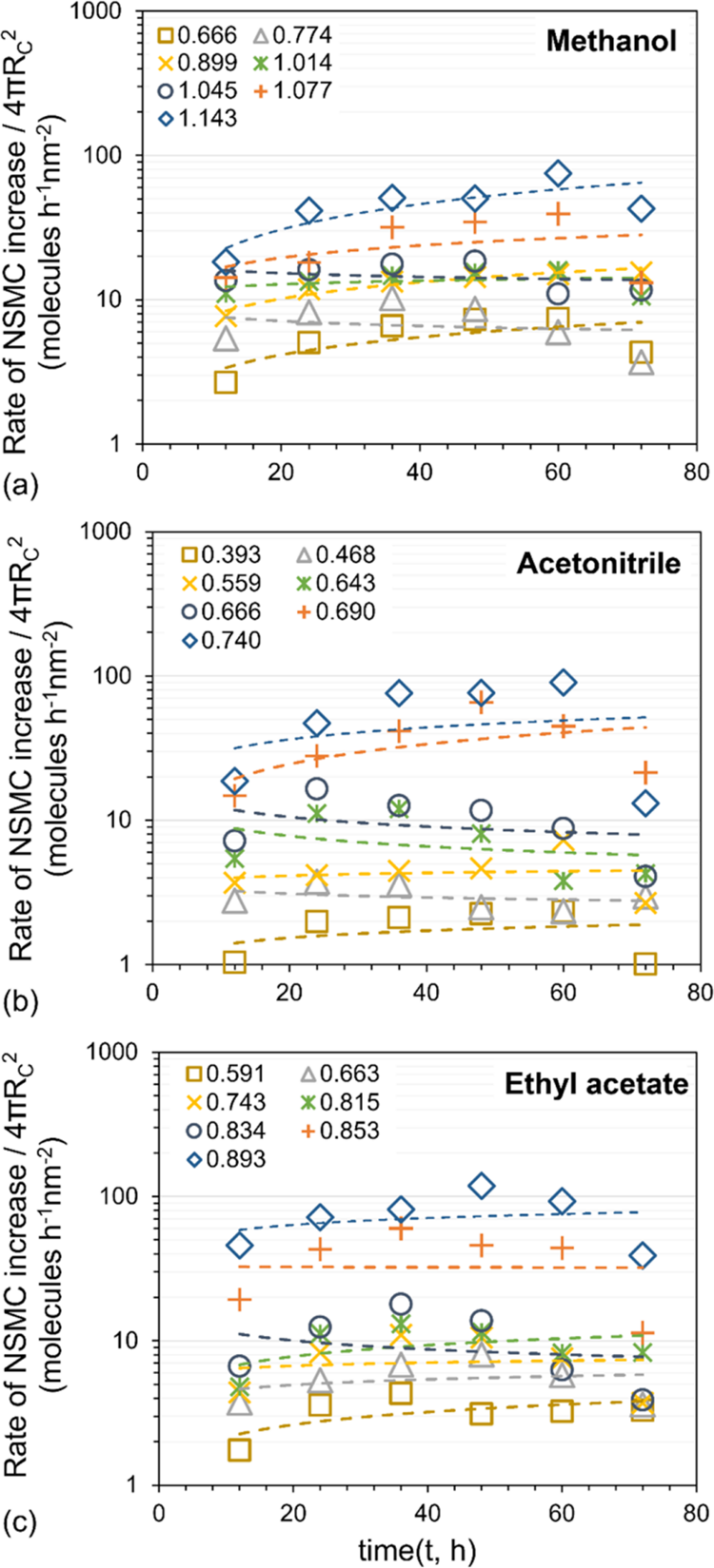
Evaluation of rate limiting process for cluster growth via interface transfer control. Rate of NSMC increase divided by cluster surface area versus time for different solution concentrations.

If the rate of increase of NSMC is governed by interface transfer where a molecule to be attached is in immediate contact with the cluster and can join by making a random jump over a distance that is comparable to the diameter of the molecule,^53^ the rate of mass flux becomes independent of the cluster radius. The rate of NSMC increase with time becomes proportional to the cluster radius squared, and the ordinate values divided by the radius squared should give horizontal lines, indicating time independence. Our results are plotted in Figure 9.

Figure 9 shows several horizontal curves and moderate variation among some curves, with the change with time much less as compared with that shown in Figure S5 for methanol. There is a weak tendency for the ordinate to increase with time, especially at the highest and the lowest concentrations but is relatively constant at intermediate concentrations. Accordingly, within the uncertainty of the data, it appears that the size increase of the clusters is governed by interface transfer control. The ordinate is increasing with increasing concentration; i.e., the rate of increase in NSMC/surface area is higher at higher concentration as expected.

In further discussing the origin and effect of the solvent–solute interactions that direct the cluster size, we note that there is a clear correlation between the shift in the high-wavenumber carbonyl (C=O) stretching peak and the MD-predicted solvation free energy values in the different solvents at 298 K (Figure S6). The peak shifts to a higher wavenumber in the order: methanol < acetonitrile < ethyl acetate and the solvation free energy values decrease in the same order, both revealing a gradually weaker solvent–solute interaction involving the C=O group in the order: methanol > acetonitrile > ethyl acetate.

### 4.2 Relation to Nucleation Behavior

Table 3 combines the cluster size determinations of the present work with data determined for the nucleation of salicylamide in previous work.^30^ The first data column in the table gives the driving force required to reach a specific nucleation induction time (2000 s) in the three solvents and is used as a measure of the nucleation propensity. The lower the value, the easier the nucleation. These values are determined directly from the data of the nucleation experiments without relying on a specific theory. The second data column gives the interfacial energy determined from by fitting the nucleation experimental data to classical nucleation theory, CNT. The third and fourth data columns are experimental data from the present work, i.e., supersaturation used and the corresponding cluster size at those supersaturations. The final column gives the critical nucleus size calculated from eq 3 for the same supersaturations.
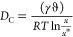
3where γ is the interfacial energy^30^ given in Table 3, ϑ is the molecular volume (0.000101853 m^3^ mol^–1^), *R* is the gas constant (8.314 J mol^–1^K^–1^), and *T* is the temperature (298 K).

**Table 3:** Clustering and Nucleation Data^a^ for Salicylamide in Different Solvents

solvent	driving force to reach τ_50_ of 2000 s (*RT* ln*S*)^b^	interfacial energy (mJ/m^2^)^c^	supersaturation (*x*/x*, mol/L/mol/L)	size of cluster after 72 h (*D*_S_, nm)	critical nucleus size (*D*_C_, nm)^d^
ethyl acetate	1006	2.67	1.02	1439	22.16
			1.07	2807	6.49
acetonitrile	1495	3.34	1.03	1387	18.57
			1.11	2101	5.26
methanol	1619	3.97	1.03	955	22.07
			1.09	1745	7.57

a Using results from ref (24) without activity coefficient correction.

b Estimated directly from the data of the nucleation experiments.

c Obtained by fitting CNT to the data of the nucleation experiments from ref (30).^30^

d Calculated using eq 3.

In Table 4, cluster sizes at *x*/x* = 1.05 are determined by linear interpolation of the data in Table 3 and are compared with the size of the critical nucleus estimated for the same driving force from the interfacial energy by using eq 3.

**Table 4:** Mean Size of Prenucleation Cluster (*D*_s_) Calculated for Supersaturation (*x*/x* = 1.05) by Linear Interpolation of Experimentally Determined *D*_s_ Values Given in Table 3 Compared with Critical Nucleus Size (*D*_c_) for Salicylamide at the Same Supersaturation Calculated Using Equation 3

solvent	size of cluster after 72 h (*D*_S_, nm)	critical nucleus size (*D*_C_, nm) (eq 3)
ethyl acetate	2046	8.99
acetonitrile	1499	11.25
methanol	1149	13.37

**Figure 10:**
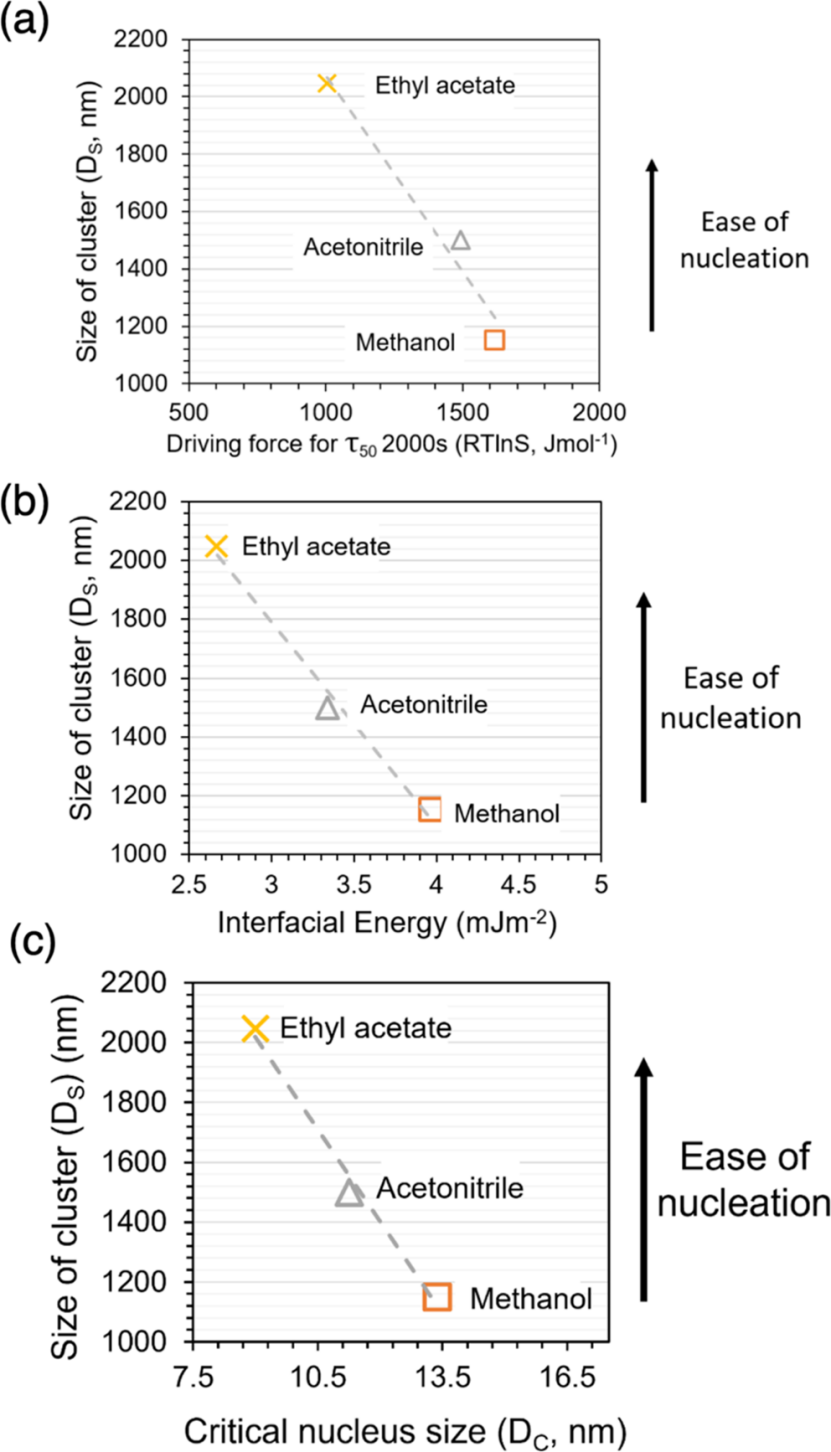
Size of salicylamide clusters (D_s_) obtained in this work after 72 h, as measured under supersaturation conditions at *x*/x* = 1.05 in the three solvents (data is given in Table 4). (a) *D*_s_ vs driving force required to reach the nucleation induction time (τ_50_) of 2000 s (Table 3); (b) *D*_s_ vs interfacial energy (Table 3); (c) *D*_s_ vs critical nucleus size (*D*_c_) (Table 4). Methanol (orange, orange box), acetonitrile (gray, gray delta), and ethyl acetate (yellow, yellow times). The arrow indicates that nucleation becomes easier in that direction.

As shown in Figure 10, we find a linear relation between the size of prenucleation clusters formed at weakly supersaturated conditions (*x*/x* = 1.05) (Table 4), and the ease of nucleation as characterized by the driving force required to reach the nucleation induction time of 2000 s (Table 3, using data from ref (30)). The plot shows how the largest clusters form in ethyl acetate where the driving force required for nucleation with an induction time of 2000 s is the lowest, i.e., the nucleation is the easiest. The smallest clusters form in methanol where the driving force required for nucleation is the highest, and the behavior in acetonitrile is in-between. These results indicate that a higher propensity for crystal nucleation of a compound in these three different solvents is associated with the formation of larger prenucleation clusters.

Since the propensity for nucleation is well captured by the interfacial energy calculated from the nucleation data using classical nucleation theory,^30^ the size of the prenucleation clusters in the supersaturated solutions at *x*/x* = 1.05 is inversely related to the interfacial energy values (Figure 10b). As shown in Figure 10c, there is also a very clear relationship across the three solvents between size of prenucleation clusters and the critical nucleus size calculated for x/x* = 1.05, Table 4. Clearly, large prenucleation clusters are found in a solvent where the solid–liquid interfacial energy is lowered, which in turn leads to small values for the critical nucleus and nucleation is easy. Within classical nucleation theory, a larger magnitude of negative solid–liquid interfacial energy means that a smaller number of molecules need to assemble to form a critical nucleus, and so nucleation becomes easier. These findings corroborate our previous work on salicylic acid and fenoxycarb.^25^ Furthermore, for all three solutes, the large prenucleation clusters and the corresponding ease of nucleation are observed in ethyl acetate, and the opposite is observed in alcohols.

**Figure 11:**
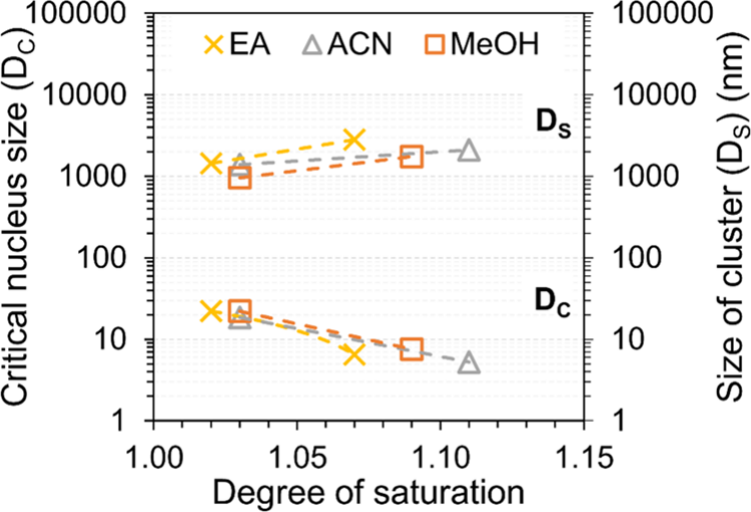
Size of prenucleation clusters (*D*_s_) obtained in this work after 72 h (right-hand scale and upper part of the data), and the critical nucleus size (*D*_c_) calculated using the previous nucleation results^30^ (left-hand scale and lower part of the data) and eq 3, in the same solvents at different supersaturation or degree of saturation. Methanol (orange, orange box), acetonitrile (gray, gray delta), and ethyl acetate (yellow, yellow times).

Figure 11 shows the relationship between the critical nucleus size, *D*_c_, as calculated by eq 3, and the size of the prenucleation clusters obtained experimentally in the present work after 72 h at different supersaturations. Notably, the critical nucleus sizes are calculated for the same supersaturations (*x*/x*) at which the size of prenucleation clusters was determined. For simplicity, interfacial energy from CNT is used to calculate the size of critical nucleus; however, it does not suggest that this crystallization system follows CNT. Figure 11 shows that at equal supersaturation, the clusters recorded experimentally in the present work are about 2 orders of magnitude larger than the critical nucleus calculated from eq 3 based on experimentally determined interfacial energies.^30^ Accordingly, it indicates that CNT was not suitable for the studied crystallization systems of this work, and it can also be conceivable that nuclei can arise initially by a local molecular structuring within a larger solute-rich cluster in accordance with the two-step nucleation theory. However, it needs to be recognized that the clusters are determined in nonagitated solutions, while the nucleation results come from agitated solutions. It is quite possible that the cluster size depends on hydrodynamics and fluid shear since nucleation does.^54^ Further, it should be recognized that besides being exposed to agitation, the nucleation experiments^30^ were performed at higher supersaturations. Accordingly, the calculation of the nucleus sizes involves an extrapolation of the nucleation data to much lower supersaturations, which gives uncertainty. Another aspect is that the induction times for crystal nucleation at the conditions of the clustering experiments in the present work would be very long, and this is of course part of the experimental design to allow investigation of the prenucleation clustering.

**Figure 12:**
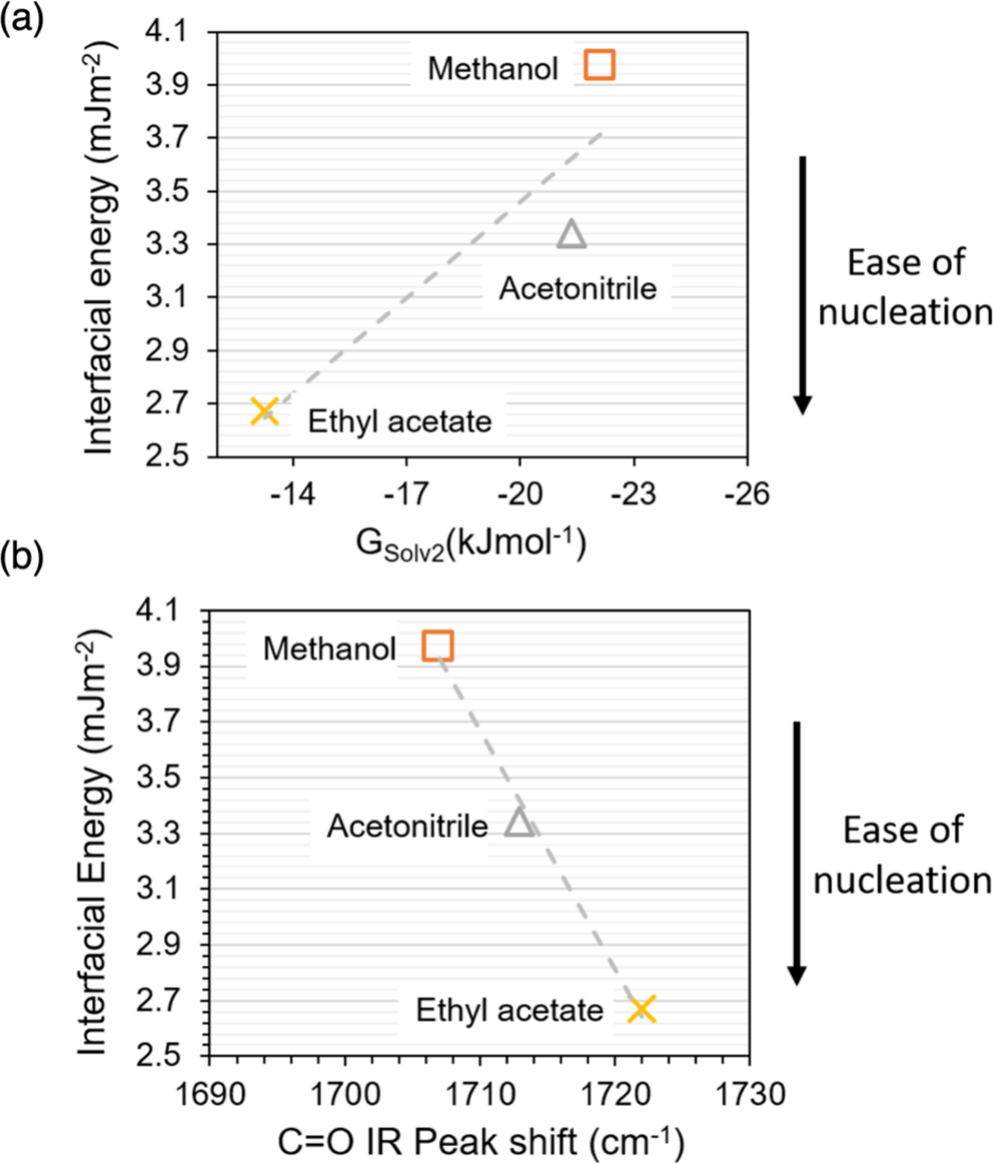
Relationship between interfacial energy calculated in previous nucleation study^30^ and (a) the free energy of solvation computed by the Poisson–Boltzmann continuum solvation method and (b) carbonyl peak shift obtained experimentally by IR spectroscopy in this work. Methanol (orange, orange box), acetonitrile (gray, gray delta), and ethyl acetate (yellow, yellow times).

In previous work on risperidone^55^ and salicylic acid,^56^ the nucleation propensity was found to correlate with the strength of solute–solvent interaction such that the weaker the solute–solvent interaction, the lower the solid–solution interfacial energy. In Figure 12, a similar trend is shown for salicylamide interfacial energy against the free energy of solvation computed by the Poisson–Boltzmann continuum solvation method and likewise for the experimentally recorded shift in the carbonyl IR peak. In a solvent for which the solvent–salicylamide interaction is weaker (calculated as a smaller magnitude for the negative solvation free energy or experimentally recorded by IR spectroscopy as a higher wavenumber for the carbonyl peak), the interfacial energy is lower and the nucleation is easier.

For salicylamide, we find that the ease of nucleation in the three solvents as determined under the conditions in previous work^30^ (higher supersaturation and in agitated solutions) is correlated to the size of the prenucleation clusters recorded in the present work by photon correlation spectroscopy in nonagitated weakly supersaturated solutions. The larger the clusters, the easier the nucleation. This further correlates with the strength of solvent–solute interactions as predicted and supported by molecular modeling in this work. This agrees with the previous results on salicylic acid and fenoxycarb clustering in different solvents^25^ and for all three solutes nucleation is easy in ethyl acetate and difficult in alcohol. Accordingly, a strong solute–solvent interaction leads to smaller prenucleation clusters and a more difficult nucleation, and so predictive screening of solvation energies is a key ingredient in the search for alternative, greener solvents that can direct desired properties of the crystallized pharmaceutical products.

## 5 Conclusions

In this work, molecular clustering of salicylamide in three different organic solvents, ethyl acetate, methanol, and acetonitrile, was investigated using photon correlation spectroscopy complemented with IR spectroscopy and atomic-scale MD simulations. The cluster size of salicylamide in ethyl acetate, acetonitrile, and methanol increases with time over a period of 72 h and increases with increasing solute concentration (from undersaturated via saturated to supersaturated solutions). The size of salicylamide clusters in a saturated solution after 72 h is just above 400 nm, roughly independent of solvent. Below saturation, the cluster size at equal time and relative concentration (x/x*) changes with the solvent in the same order as the solubility: acetonitrile < ethyl acetate < methanol. The cluster size at low supersaturation (*x*/x* = 1.05) in the unstirred solution is clearly correlated to ease of nucleation at high supersaturation in agitated solutions. The larger the prenucleation clusters, the easier the nucleation. The strength of solute–solvent interaction computed and rationalized from molecular models is well correlated with the wavenumber shift of the carbonyl peak as recorded by IR spectroscopy. The solute–solvent interaction is weaker in ethyl acetate, intermediate in acetonitrile, and stronger in methanol, and this interaction strength is accordingly correlated with size of prenucleation clusters and ease of crystal nucleation. In previous work,^30^ we have suggested that a reduced rate of desolvation is the reason why crystal nucleation becomes more difficult as the solute–solvent interaction becomes stronger. However, within the framework of the two-step nucleation theory,^7^ the clustering data of the present work and of the previous work on fenoxycarb and salicylic acid^30^ suggest that the direct cause for this relation may in fact be stronger solvation leading to smaller prenucleation clusters, and these smaller clusters make nucleation more difficult.

## Supporting Information Available

Details of experimental methodology for clustering and viscosity experiments, fitting Lennard-Jones (LJ) force field parameters using GULP, alchemical free energy method, and Poisson–Boltzmann continuum solvation method; image explaining the thermodynamic integration model of solvation free energy model; graphs of initial and final structures of modeling experiments and clustering behavior of salicylamide in three different solvents using optimized interatomic LJ potentials obtained with GULP; graph for evaluation of rate limiting process for cluster growth–diffusion control; and comparison of carbonyl stretching peak vs the solvation free energy values (PDF)

## References

[ref1] Cofman V. The Scientific Papers of Willard Gibbs. Science 1927, 66, 510 10.1126/science.66.1717.510. 17771623

[ref2] Frenkel J. Statistical Theory of Condensation Phenomena. J. Chem. Phys. 1939, 7 (3), 200–201. 10.1063/1.1750413.

[ref3] Oppermann R. H. Kinetik der phasenbildung. J. Franklin Inst. 1940, 229, 539 10.1016/s0016-0032(40)90639-1.

[ref4] Zeldovich Y. B. 10. On the Theory of New Phase Formation. Cavitation. In Selected Works of Yakov Borisovich Zeldovich, Volume I; DE Gruyter, 2015; Vol. 18, pp 120–137.

[ref5] Volmer M.; Weber A. Keimbildung in Übersättigten Gebilden. Z. Phys. Chem. 1926, 119U (1), 277–301. 10.1515/zpch-1926-11927.

[ref6] Mahajan A. S. Electric Field. Phys. Educ. 1980, 15, 67 10.1088/0031-9120/15/2/102.

[ref7] Ten Wolde P. R.; Frenkel D. Enhancement of Protein Crystal Nucleation by Critical Density Fluctuations. Science 1997, 277 (5334), 1975–1978. 10.1126/science.277.5334.1975. 9302288

[ref8] Erdemir D.; Lee A. Y.; Myerson A. S. Nucleation of Crystals from Solution: Classical and Two-Step Models. Acc. Chem. Res. 2009, 42 (5), 621–629. 10.1021/ar800217x. 19402623

[ref9] Igarashi K.; Azuma M.; Kato J.; Ooshima H. Initial Stage of Crystallization of Lysozyme: A Differential Scanning Calorimetric (DSC) Study. J. Cryst. Growth 1999, 204 (1), 191–200. 10.1016/S0022-0248(99)00181-5.

[ref10] Phillies G. D. J. Comment on “Critical Behavior of a Binary Mixture of Protein and Salt Water. Phys. Rev. Lett. 1985, 55 (12), 1341 10.1103/PhysRevLett.55.1341. 10031792

[ref11] Georgalis Y.; Umbach P.; Raptis J.; Saenger W. Lysozyme Aggregation Studied by Light Scattering. II. Variations of Protein Concentration. Acta Crystallogr., Sect. D: Biol. Crystallogr. 1997, 53 (6), 703–712. 10.1107/S0907444997006859. 15299858

[ref12] Vogtt K.; Javid N.; Alvarez E.; Sefcik J.; Bellissent-Funel M. C. Tracing Nucleation Pathways in Protein Aggregation by Using Small Angle Scattering Methods. Soft Matter 2011, 7 (8), 3906–3914. 10.1039/c0sm00978d.

[ref13] Sedlák M. Large-Scale Supramolecular Structure in Solutions of Low Molar Mass Compounds and Mixtures of Liquids: II. Kinetics of the Formation and Long-Time Stability. J. Phys. Chem. B 2006, 110 (9), 4339–4345. 10.1021/jp056934x. 16509732

[ref14] Sedlák M. Large-Scale Supramolecular Structure in Solutions of Low Molar Mass Compounds and Mixtures of Liquids: I. Light Scattering Characterization. J. Phys. Chem. B 2006, 110 (9), 4329–4338. 10.1021/jp0569335. 16509731

[ref15] Sedlák M. Large-Scale Supramolecular Structure in Solutions of Low Molar Mass Compounds and Mixtures of Liquids. III. Correlation with Molecular Properties and Interactions. J. Phys. Chem. B 2006, 110 (28), 13976–13984. 10.1021/jp061919t. 16836350

[ref16] Vekilov P. G.; Vorontsova M. A. Nucleation Precursors in Protein Crystallization. Acta Crystallogr., Sect. F: Struct. Biol. Commun. 2014, 70 (3), 271–282. 10.1107/S2053230X14002386. 24598910 PMC3944685

[ref17] Jawor-Baczynska A.; Moore B. D.; Lee H. S.; McCormick A. V.; Sefcik J. Population and Size Distribution of Solute-Rich Mesospecies within Mesostructured Aqueous Amino Acid Solutions. Faraday Discuss. 2014, 167 (0), 425–440. 10.1039/c3fd00066d. 24640504

[ref18] Hagmeyer D.; Ruesing J.; Fenske T.; Klein H. W.; Schmuck C.; Schrader W.; da Piedade M. E. M.; Epple M. Direct Experimental Observation of the Aggregation of α-Amino Acids into 100–200 Nm Clusters in Aqueous Solution. RSC Adv. 2012, 2 (11), 4690–4696. 10.1039/c2ra01352e.

[ref19] Barua H.; Svärd M.; Rasmuson Å. C.; Hudson S. P.; Cookman J. Mesoscale Clusters in the Crystallisation of Organic Molecules. Angew. Chem., Int. Ed. 2024, 63 (10), e202312100 10.1002/anie.202312100. 38055699

[ref20] Huang Y.; Wang N.; Wang J.; Ji X.; Yang J.; Huang X.; Zhou L.; Wang T.; Hao H. Regulating of Liquid–Liquid Phase Separation and Molecular Self-Assembly through Selective Solvation. Ind. Eng. Chem. Res. 2023, 62 (47), 20459–20469. 10.1021/acs.iecr.3c02796.

[ref21] Zong S.; Wang J.; Huang X.; Wang T.; Liu Q.; Tian B.; Xie C.; Hao H. Molecular Evolution Pathways during Nucleation of Small Organic Molecules: Solute-Rich Pre-Nucleation Species Enable Control over the Nucleation Process. Phys. Chem. Chem. Phys. 2020, 22 (33), 18663–18671. 10.1039/D0CP03493B. 32794537

[ref22] Sorensen T. J.; Sontum P. C.; Samseth J.; Thorsen G.; Malthe-Sorenssen D. Cluster Formation in Precrystalline Solutions. Chem. Eng. Technol. 2003, 26 (3), 307–312. 10.1002/ceat.200390047.

[ref23] Tsarfati Y.; Biran I.; Wiedenbeck E.; Houben L.; Cölfen H.; Rybtchinski B. Continuum Crystallization Model Derived from Pharmaceutical Crystallization Mechanisms. ACS Cent. Sci. 2021, 7 (5), 900–908. 10.1021/acscentsci.1c00254. 34079905 PMC8161475

[ref24] Cookman J.; Hamilton V.; Hall S. R.; Bangert U. Non-Classical Crystallisation Pathway Directly Observed for a Pharmaceutical Crystal via Liquid Phase Electron Microscopy. Sci. Rep. 2020, 10 (1), 19156 10.1038/s41598-020-75937-2. 33154480 PMC7644682

[ref25] Kakkar S.; Devi K. R.; Rasmuson Å. C. Molecular Clustering of Fenoxycarb and Salicylic Acid in Organic Solvents and Relation to Crystal Nucleation. Cryst. Growth Des. 2022, 22 (5), 2824–2836. 10.1021/acs.cgd.1c00913.

[ref26] Sasada Y.; Takano T.; Kakudo M. Crystal Structure of Salicylamide. Bull. Chem. Soc. Jpn. 1964, 37 (7), 940–946. 10.1246/bcsj.37.940.

[ref27] Johnstone R. D. L.; Lennie A. R.; Parker S. F.; Parsons S.; Pidcock E.; Richardson P. R.; Warren J. E.; Wood P. A. High-Pressure Polymorphism in Salicylamide. CrystEngComm 2010, 12 (4), 1065–1078. 10.1039/b921288d.

[ref28] Nordström F. L.; Rasmuson Å. C. Solubility and Melting Properties of Salicylamide. J. Chem. Eng. Data 2006, 51 (5), 1775–1777. 10.1021/je060178m.

[ref29] Nordström F. L.; Svärd M.; Rasmuson Å. C. Primary Nucleation of Salicylamide: The Influence of Process Conditions and Solvent on the Metastable Zone Width. CrystEngComm 2013, 15 (36), 7285–7297. 10.1039/c3ce40619a.

[ref30] Kakkar S.; Devi K. R.; Svärd M.; Rasmuson Å. Crystal Nucleation of Salicylamide and a Comparison with Salicylic Acid. CrystEngComm 2020, 22, 3329–3339. 10.1039/D0CE00168F.

[ref31] Lynch A.; Jia L.; Svärd M.; Rasmuson Å. C. Crystal Growth of Salicylamide in Organic Solvents. Cryst. Growth Des. 2018, 18 (12), 7305–7315. 10.1021/acs.cgd.8b00767.

[ref32] Vanommeslaeghe K.; MacKerell A. D. Jr Automation of the CHARMM General Force Field (CGenFF) I: Bond Perception and Atom Typing. J. Chem. Inf. Model. 2012, 52 (12), 3144–3154. 10.1021/ci300363c. 23146088 PMC3528824

[ref33] Groom C. R.; Bruno I. J.; Lightfoot M. P.; Ward S. C. The Cambridge Structural Database. Acta Crystallogr., Sect. B: Struct. Sci., Cryst. Eng. Mater. 2016, 72 (2), 171–179. 10.1107/S2052520616003954. PMC4822653 27048719

[ref34] Phetmung H.; Musikapong K.; Srichana T. Thermal Analysis, Structure, Spectroscopy and DFT Calculations of a Pharmaceutical Cocrystal of Salicylic Acid and Salicylamide. J. Therm. Anal. Calorim. 2019, 138, 1207–1220. 10.1007/s10973-019-08794-5.

[ref35] Gale J. D. GULP: A Computer Program for the Symmetry-Adapted Simulation of Solids. J. Chem. Soc., Faraday Trans. 1997, 93 (4), 629–637. 10.1039/a606455h.

[ref36] Huang J.; Rauscher S.; Nawrocki G.; Ran T.; Feig M.; de Groot B. L.; Grubmüller H.; MacKerell A. D. CHARMM36m: An Improved Force Field for Folded and Intrinsically Disordered Proteins. Nat. Methods 2017, 14 (1), 71–73. 10.1038/nmeth.4067. 27819658 PMC5199616

[ref37] Berendsen H. J. C.; van der Spoel D.; van Drunen R. GROMACS: A Message-Passing Parallel Molecular Dynamics Implementation. Comput. Phys. Commun. 1995, 91 (1–3), 43–56. 10.1016/0010-4655(95)00042-E.

[ref38] Kutzner C.; Páll S.; Fechner M.; Esztermann A.; de Groot B. L.; Grubmüller H. More Bang for Your Buck: Improved Use of GPU Nodes for GROMACS 2018. J. Comput. Chem. 2019, 40 (27), 2418–2431. 10.1002/jcc.26011. 31260119

[ref39] Swope W. C.; Andersen H. C.; Berens P. H.; Wilson K. R. A Computer Simulation Method for the Calculation of Equilibrium Constants for the Formation of Physical Clusters of Molecules: Application to Small Water Clusters. J. Chem. Phys. 1982, 76 (1), 637–649. 10.1063/1.442716.

[ref40] Hess B.; Bekker H.; Berendsen H. J. C.; Fraaije J. G. E. M. LINCS: A Linear Constraint Solver for Molecular Simulations. J. Comput. Chem. 1997, 18 (12), 1463–1472. 10.1002/(SICI)1096-987X(199709)18:12<1463::AID-JCC4>3.0.CO;2-H.

[ref41] Miyamoto S.; Kollman P. A. Settle: An Analytical Version of the SHAKE and RATTLE Algorithm for Rigid Water Models. J. Comput. Chem. 1992, 13 (8), 952–962. 10.1002/jcc.540130805.

[ref42] Darden T.; York D.; Pedersen L. Particle Mesh Ewald: An N· Log (N) Method for Ewald Sums in Large Systems. J. Chem. Phys. 1993, 98 (12), 10089–10092. 10.1063/1.464397.

[ref43] Bussi G.; Donadio D.; Parrinello M. Canonical Sampling through Velocity Rescaling. J. Chem. Phys. 2007, 126 (1), 014101 10.1063/1.2408420. 17212484

[ref44] Berendsen H. J. C.; Postma J. P. M.; van Gunsteren W. F.; DiNola A.; Haak J. R. Molecular Dynamics with Coupling to an External Bath. J. Chem. Phys. 1984, 81 (8), 3684–3690. 10.1063/1.448118.

[ref45] Parrinello M.; Rahman A. Polymorphic Transitions in Single Crystals: A New Molecular Dynamics Method. J. Appl. Phys. 1981, 52 (12), 7182–7190. 10.1063/1.328693.

[ref46] Shirts M. R.; Mobley D. L.; Chodera J. D. Chapter 4 Alchemical Free Energy Calculations: Ready for Prime Time?. Annu. Rep. Comput. Chem. 2007, 3, 41–59. 10.1016/S1574-1400(07)03004-6.

[ref47] Xu L.; Sun H.; Li Y.; Wang J.; Hou T. Assessing the Performance of MM/PBSA and MM/GBSA Methods. 3. The Impact of Force Fields and Ligand Charge Models. J. Phys. Chem. B 2013, 117 (28), 8408–8421. 10.1021/jp404160y. 23789789

[ref48] Kollman P. A.; Massova I.; Reyes C.; Kuhn B.; Huo S.; Chong L.; Lee M.; Lee T.; Duan Y.; Wang W.; et al. Calculating Structures and Free Energies of Complex Molecules: Combining Molecular Mechanics and Continuum Models. Acc. Chem. Res. 2000, 33 (12), 889–897. 10.1021/ar000033j. 11123888

[ref49] Takac M. J.-M.; Topić D. V. FT-IR and NMR Spectroscopic Studies of Salicylic Acid Derivatives. II. Comparison of 2-Hydroxy- and 2,4- and 2,5-Dihydroxy Derivatives. Acta Pharm. 2004, 54 (3), 177–191. 15610615

[ref50] Svärd M.; Devi K. R.; Khamar D.; Mealey D.; Cheuk D.; Zeglinski J.; Rasmuson Å. C. Solute Clustering in Undersaturated Solutions-Systematic Dependence on Time, Temperature and Concentration. Phys. Chem. Chem. Phys. 2018, 20 (22), 15550–15559. 10.1039/C8CP01509K. 29808866

[ref51] Kakkar S.; Devi K. R.; Rasmuson Å. Nanoclusters of Fenoxycarb and Salicylic Acid in Different Organic Solvents: Correlation with Nucleation Behaviour In Preparation.

[ref52] Bonnett P. E.; Carpenter K. J.; Dawson S.; Davey R. J. Solution Crystallisation via a Submerged Liquid-Liquid Phase Boundary: Oiling Out. Chem. Commun. 2003, 3 (6), 698–699. 10.1039/b212062c. 12703779

[ref53] Kashchiev D.; van Rosmalen G. M. Review: Nucleation in Solutions Revisited. Cryst. Res. Technol. 2003, 38 (78), 555–574. 10.1002/crat.200310070.

[ref54] Liu J.; Rasmuson Å. C. Influence of Agitation and Fluid Shear on Primary Nucleation in Solution. Cryst. Growth Des. 2013, 13 (10), 4385–4394. 10.1021/cg4007636.

[ref55] Mealey D.; Zeglinski J.; Khamar D.; Rasmuson Å. C. Influence of Solvent on Crystal Nucleation of Risperidone. Faraday Discuss. 2015, 179, 309–328. 10.1039/C4FD00223G. 25886651

[ref56] Khamar D.; Zeglinski J.; Mealey D.; Rasmuson Å. C. Investigating the Role of Solvent-Solute Interaction in Crystal Nucleation of Salicylic Acid from Organic Solvents. J. Am. Chem. Soc. 2014, 136 (33), 11664–11673. 10.1021/ja503131w. 25029039

