# Supplementary material for: Tracking Prenucleation Molecular Clustering of Salicylamide in Organic Solvents
Source: Cryst Growth Des. 2024 Jun 21;24(13):5740–53. doi: 10.1021/acs.cgd.4c00507 (PMC11228918; doi:10.1021/acs.cgd.4c00507)
Supplement: Supplementary file 1 — cg4c00507_si_001.pdf [file cg4c00507_si_001.pdf]

Supplementary Information

Tracking pre-nucleation molecular clustering of salicylamide in organic solvents

Shubhangi Kakkar[¥1], *Shayon Bhattacharya*[¥2], *Pierre-André Cazade*[2], *Damien Thompson*[*1,2], *Åke Rasmuson*[*1,3]

[1]*Department of Chemical Sciences, Bernal Institute, University of Limerick, Limerick, Ireland.*

[2]*Department of Physics, Bernal Institute, University of Limerick, Limerick, Ireland.*

[3]*Department of Chemical Engineering and Technology, KTH Royal Institute of Technology, Stockholm, Sweden.*

[¥]These authors contributed equally to this work.

* *Email: Ake.Rasmuson@ul.ie, Damien,Thompson@ul.ie*

Table of Contents

Supplementary Notes **Pages**

S1. Cluster size determinations ……………………………………………………………S3

S2. Viscosity Measurements ……………………………………………………………...S3

S3. Fitting Lennard-Jones (LJ) force field parameters using GULP…………………………S3

S4. Alchemical free energy method of estimating solubility...S5

S5. Poisson–Boltzmann continuum solvation method S5

Figures

Fig. S1. Thermodynamic integration model of solvation free energy ………………………..S7

Fig. S2. Molecular dynamics simulations (500 ns) of salicyamide molecules using standard forcefield parameters in three solvents, methanol, ethyl acetate and acetonitrile ……………………………………………………………………………………..…S8

Fig. S3. Molecular dynamics simulations of 12-mer, 24-mer, 48-mer, and 1600-mer salicylamide molecules in a crystal polymorph in methanol, ethyl acetate, acetonitrile and water using standard forcefield parameters …………………………………………………..…S9

Fig. S4. Clustering behaviour of salicylamide in methanol (MeOH), ethyl acetate (EtAc) and acetonitrile (AcN) using optimised interatomic potentials obtained with GULP…………S10

Fig. S5. Evaluation of rate limiting process for cluster growth – diffusion control. Rate of NSMC increase divided by cluster radius versus time for different solution concentrations...S11

Fig. S6. Shift in carbonyl stretching peak *vs.* the solvation free energy values obtained using the methods described above in the three solvents, methanol, acetonitrile and ethyl acetate..S12

Supplementary References…………………………………………………………..S12

Supplementary Notes

S1. Cluster size determinations

Solvodynamic diameter measurements were performed in a *Malvern Zetasizer ZSP Nano* instrument, equipped with a temperature controller. The instrument works on photon correlation spectroscopy (PCS) technology also known as Quasi-Elastic Light Scattering. Malvern *Zetasizer software v. 7.11* was used to analyse the PCS data. In every 3 hours, with the help of pipette 1500 µL the previously prepared sample was carefully added to 12 mm square PCS glass cuvette with a path length of 10 mm. An equilibration time of 120 seconds was given at 298K for all the solutions before the measurements. In each concentration at each time, 5 scans were performed. The measurements were carried out for 72 hours at isothermal conditions. The measurements were done with a forward scattering angle of 12.8°. Laser light of wavelength 632.8 nm with automatic cell positioning and an automatic attenuator were used for the measurements. Multiple narrow modes were used to calculate the intensity size distributions from autocorrelation function. Nucleation was visible under the microscope for methanol and ethyl acetate after 72 hours whereas not for acetonitrile samples.

S2. Viscosity Measurements

Viscosity measurements were performed in *Brookfield DV3TRVTJ Rheometer* prior to PCS measurements. All the measurements were performed at clustering temperature. An internal spring was used especially for low viscosity systems SC4/18. The temperature of the measurements was controlled by using a water-jacketed small volume cell which connected to a water bath and a thermocouple. The values were measured in centipoise. For samples with methanol as the solvent, measurements were performed at a speed of 180 rpm, with ethyl acetate as the solvent, measurements were performed at a speed of 150 rpm and with acetonitrile as the solvent, measurements were performed at a speed of 190 rpm. All measurements were performed for 1 min and the results obtained had an accuracy of ±1.88%.

S3. Fitting Lennard-Jones (LJ) force field parameters using GULP

In order to identify the clustering behaviour of salicylamide in three different solvents, methanol (MeOH), ethyl acetate (EtAc) and acetonitrile (AcN), we first predicted partial charges and parameters for salicylamide using CHARMM General Force Field (CGenFF)[1,2]. Our initial MD simulation to mimic superstructure formation of salicylamide in the above solvents showed no instance of clustering after 500 ns long free MD (see **Fig. S2a–f** for initial

and final snapshots of the simulation box). The total number of clusters (**Fig. S2g**) and maximum number of clusters (**Fig. S2h**) formed as a function of simulation time did not reveal a decrease in number (more molecules of salicylamide form clusters, so a smaller number of clusters) with simulation time. Similarly, the most frequently formed cluster size showed cluster formation with two molecules (**Fig. S2i**) and the population of clusters formed with larger number of molecules was almost insignificant in all three solvents.

To investigate if the formation of superstructures with salicylamide requires longer MD sampling time than 500 ns, we modelled the stabilities (through MD simulations in different environments) of a crystal polymorph structure of salicylamide (see **Fig. S3a**) obtained experimentally and deposited in the Cambridge Crystallographic Data Centre[3] (CCDC code 1545142[4]; Space Group: P 2_1/c, Cell: a = 6.543 Å, b = 15.569 Å, c = 7.104 Å, α = 90.00°, β = 113.646°, γ = 90.00°). We observe that all salicylamide *n-mer crystals* (12-mer, 24-mer, 48-mer and 1600-mer) lose their crystalline integrity within the first 10 ns of dynamics in MeOH, EtAc, AcN, water and vacuum (**Fig. S3**) and break up into their monomers and smaller n-mers. The immediate destabilisation of the salicylamide crystals in different solvent environments indicated that the Lennard Jones (LJ) parameters used in our CGenFF force field to represent a single isolated molecule of salicylamide may not be representative of the interatomic potentials that drives their assembly to a superstructure or a crystalline form, which is supposed to lead to high structural stability[5].

Following the above observation, we set out to extract interatomic LJ parameters of salicylamide in the salicylamide crystal polymorph (**Fig. S3a**). We used the "General Utility Lattice Program" (GULP[6]) to model the atomic LJ potentials of salicylamide crystal unit cell. GULP is a program for simulation of molecules, clusters, polymers, surfaces, and periodic solids. The focus of GULP code is on analytical solutions, using lattice dynamics. A variety of force fields can be used within GULP including the one used in this study, CHARMM36m[7]. Therefore, after obtaining the LJ parameters with GULP in the salicylamide crystal form, we fit or optimised the parameters for a single molecule of salicylamide by performing a relaxed fitting[8]. Our new sets of molecular simulations (500 ns each) with the optimised interatomic potentials of 125 salicylamide molecules under experimentally saturated conditions reveal clustering in MeOH solvent and superstructure formation in both EtAc and AcN (see **Fig. 7** in main text and **Fig. S4**).

S4. Alchemical free energy method

In this method, the path for the movement of solute from solution to the gas phase is constructed by mapping the perturbation energies of intermediate states[9,10]. The solvation free energy is calculated using the thermodynamic integration model (illustrated in **Fig. S1**), by employing a coupling parameter λ that incorporates incremental intermolecular interactions in a stepwise manner. At each coupling step through simulation, a derivative of solvation free energy ($\Delta G_{solvation}$) is calculated as shown in **Fig. S1** and using these derivates, the solvation free energy ($G_{solvation}$) is obtained as an integral.

The solvation free energy was calculated using the thermodynamic integration method in Forcite, a molecular dynamics module of *Materials Studio software v7.0*. The structure of salicylamide and the solvent molecules were optimized using the Compass II force field[11]. Three different amorphous cells were constructed with 1 atom of salicylamide in each and 114 methanol atoms, 48 ethyl acetate atoms, 89 acetonitrile respectively. The solvent density and volume were kept constant throughout the reaction by incorporating different number of solvent molecules to create amorphous cells. The cells were equilibrated in the NVT ensemble[12] using the Forcite module. The solvation free energy values at 298 K were then calculated via thermodynamic integration, performed with 5 intermediates with the coupling parameter running from 0 to 1. For each intermediate, 10000 equilibration steps and 100000 production steps were performed. The electrostatic interaction for all solvents was calculated with an accuracy of 0.01 kcal mol^{-1} and 0.5 Å buffer width. For all van der Waals interaction calculations, a cut-off distance of 9.5 Å was used with a long-range correction. The thermostat used was Nóse-Hoover-Langevin (NHL)[13] with a Q-ratio of 1 corresponding to a relaxation time of 1 ps.

S5. Poisson–Boltzmann continuum solvation method

The end-point conformational energies were calculated from the molecular dynamics (MD) trajectories one salicylamide molecule in different solvents (methanol, acetonitrile and ethyl acetate) for 100 ns each performed using the Gromacs 2018[14,15] code with an integration time step of 2 fs, Snapshots saved every 20 ps were used for estimating the solvation free energy (Gsolvation). EMM/PBSA or ETotal was calculated using the molecular mechanics energies combined with the Poisson–Boltzmann continuum solvation (MM/PBSA) method[16,17] using the g_mmpbsa[18] package integrated in Gromacs, which can be expressed as

$$E_{MM/PBSA}(or\ E_{Total}) = E_{MM} + G_{Solvation} \quad \text{(S1)}$$

where E_{MM} is the molecular mechanics potential energy, which could further be broken down as:

$$E_{MM} = E_{Bonded} + E_{Van\,der\,Waals} + E_{Electrostatic} \qquad \text{(S2)}$$

where E_{Bonded} is the covalent bonded interaction energy including bond, angle and dihedral energy terms, $E_{Van\ der\ Waals}$ is the van der Waals interactions described by the Lennard-Jones potential function, and $E_{Electrostatic}$ is the electrostatic interactions described by the Coulomb potential function. $G_{Solvation}$ is the solvation free energy comprised of two terms[19,20] expressed as:

$$G_{Solvation} = G_{Polar} + G_{Apolar} \qquad \text{(S3)}$$

where G_{Polar} is the electrostatic contribution to solvation and is obtained by solving the Poisson-Boltzmann (PB) equation[21]; G_{Apolar} is the nonpolar contribution and is estimated from a linear relation to solvent accessible surface area (SASA) as:

$$G_{Apolar} = \gamma.SASA + b \qquad \text{(S4)}$$

where γ is a coefficient set to the surface tension of the solvent and b is a fitting parameter. The known dielectric constants[22] were set to 32.7 for methanol, 6.02 for ethyl acetate, 37.5 for acetonitrile at the clustering temperature of 25°C.

Supplementary Figures

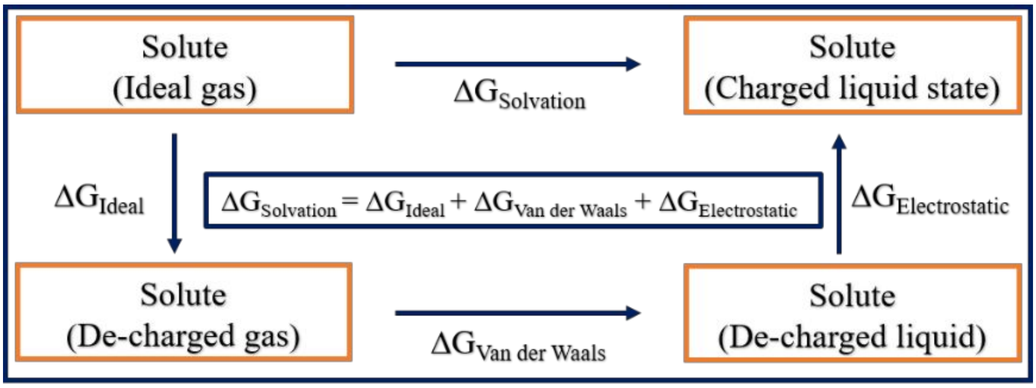

Figure S1. Thermodynamic integration model of solvation free energy.

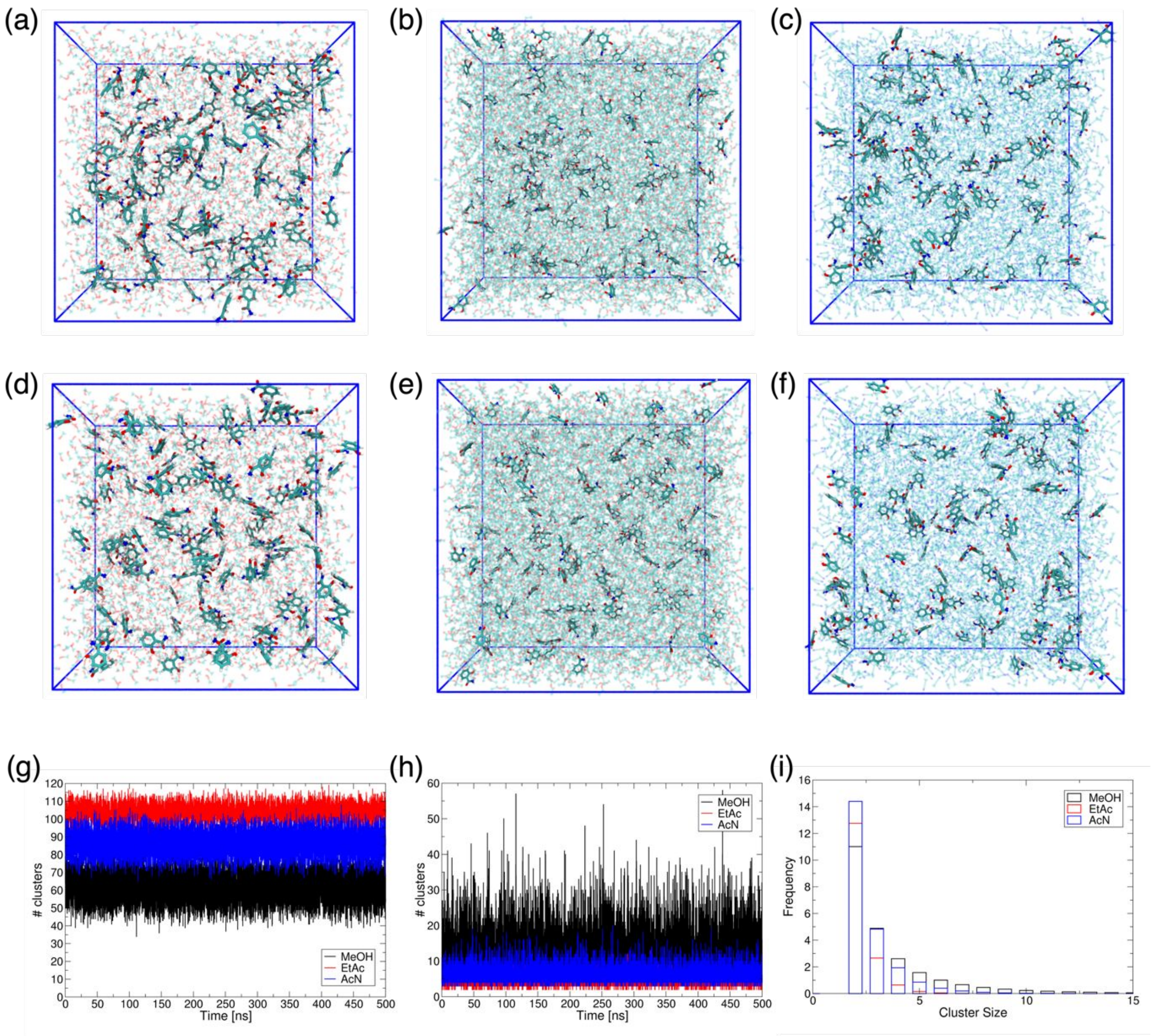

Figure S2. The starting random (**a, b, c**) and final (**d, e, f**) structures after 500 ns of free dynamics of salicylamide molecules (with CGenFF parameters) in three solvents: (**a, d**) methanol (MeOH), (**b, e**) ethyl acetate (EtAc) and (c, f) acetonitrile (AcN) shows no specific cluster formation. Timelines of the **(g)** number of clusters, **(h)** maximum number of clusters formed at a given time point, and **(i)** cluster size distribution of salicylamide in three different organic solvents.

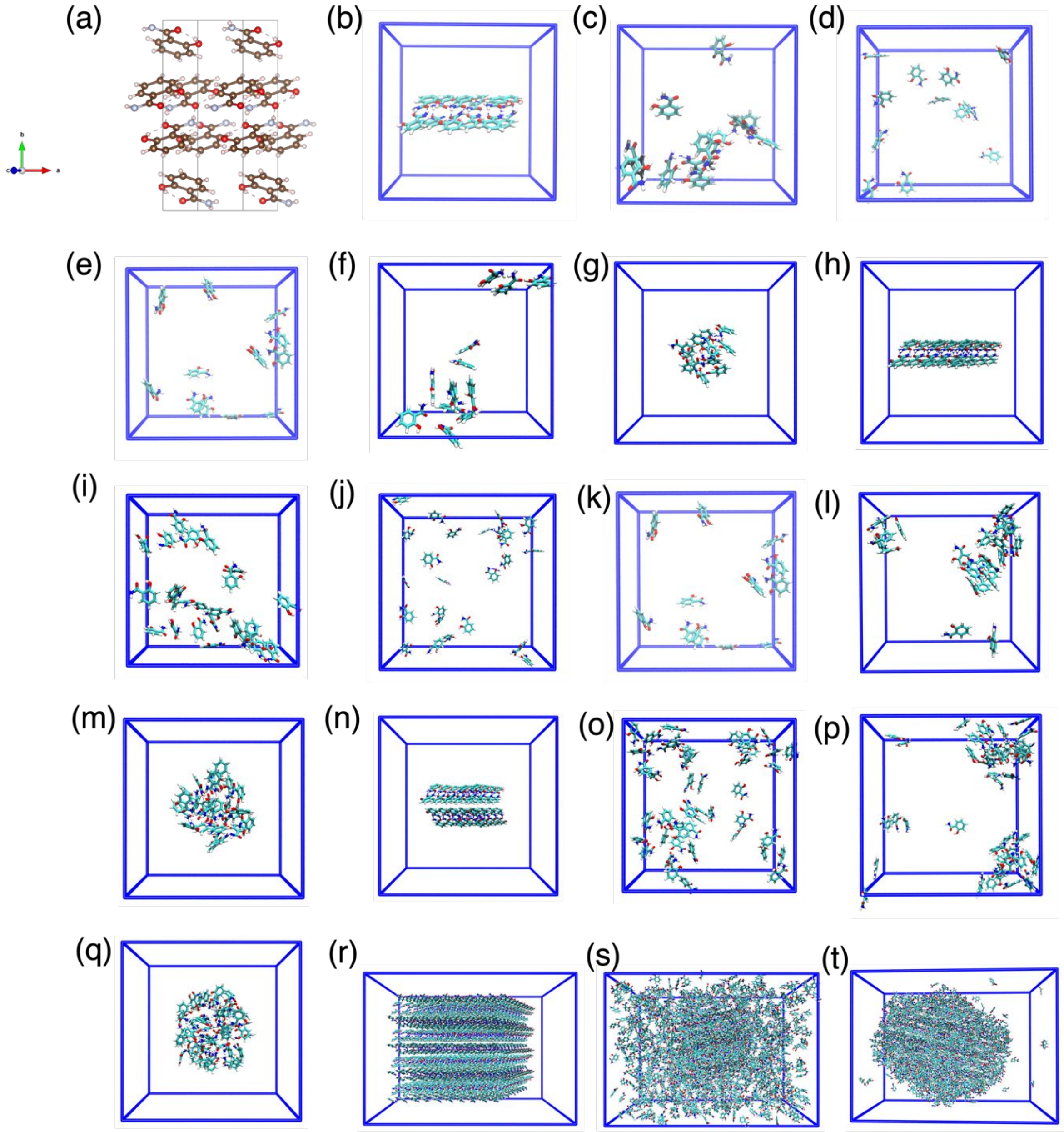

Figure S3. **(a)** The unit cell of the salicylamide crystal polymorph (CCDC code 1545142[3]used in this study to derive the interatomic LJ potentials with GULP[6] program. Starting crystal structures of **(b)** 12-mer, **(h)** 24-mer, **(n)** 48-mer, and **(r)** 1600-mer of salicylamide molecules in the polymorph. Final snapshots of **(c-g)** 12-mer salicylamide crystal structure destabilised (after 2-10 ns MD) in **(c)** MeOH, **(d)** EtAc, **(e)** AcN, **(f)** water, and **(g)** vacuum, **(i-m)** 24-mer salicylamide crystal structure destabilised in **(i)** MeOH, **(j)** EtAc, **(k)** AcN, **(l)** water, and **(m)** vacuum, **(o-q)** 48-mer salicylamide crystal structure destabilised in **(o)** MeOH, **(p)** water, and **(q)** vacuum, and **(s-t)** 1600-mer salicylamide crystal structure destabilised in **(s)** MeOH and **(t)** water.

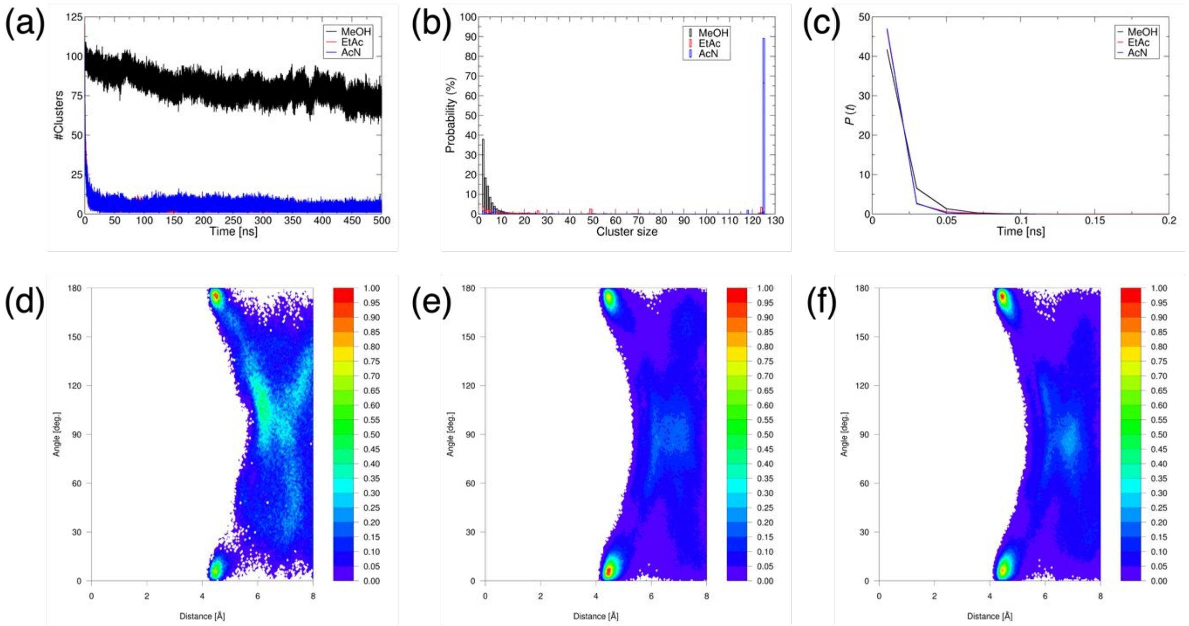

Figure S4. Clustering behaviour of salicylamide in three different solvents, methanol (MeOH), ethyl acetate (EtAc) and acetonitrile (AcN) using optimised interatomic LJ potentials obtained with GULP. **(a)** The number of clusters formed as a function of simulation time in different solvents. A second order fit of the data is provided in Fig. 7d **(b)** The percentage probability of cluster formation as function of cluster size. **(c)** Comparison of the lifetime (*P*(t)) of salicylamide–solvent H-bonds. **(d–f)** The free energy landscape of π−π stacking of salicylamide molecules (angle *Vs.* distance plots) for the full 500 ns dynamics in **(d)** MeOH, **(e)** EtAc, and **(f)** AcN. The π−π maps of the first 10 ns of initial cluster formation are provided in Figs. 9 (j–l).

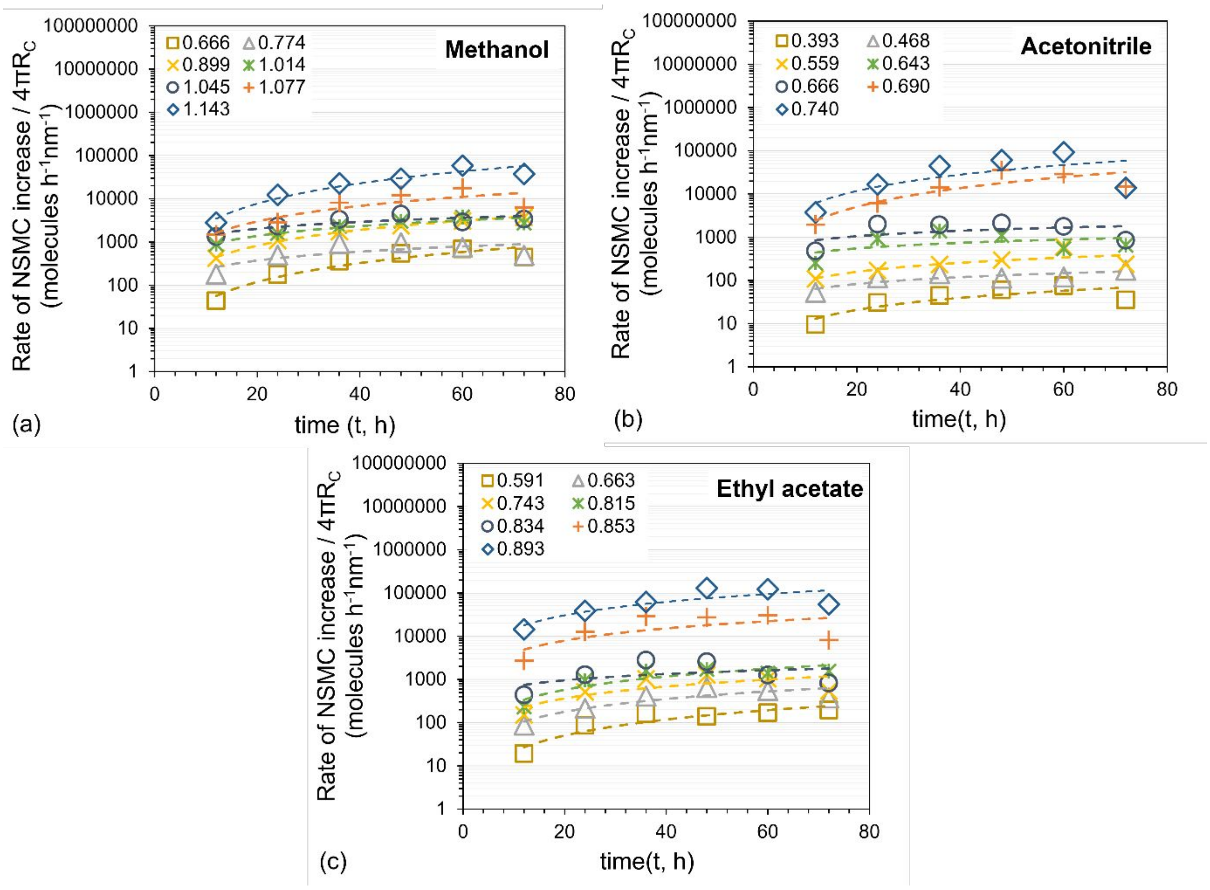

Figure S5. Evaluation of rate limiting process for cluster growth – diffusion control. Rate of NSMC increase divided by cluster radius versus time for different solution concentrations.

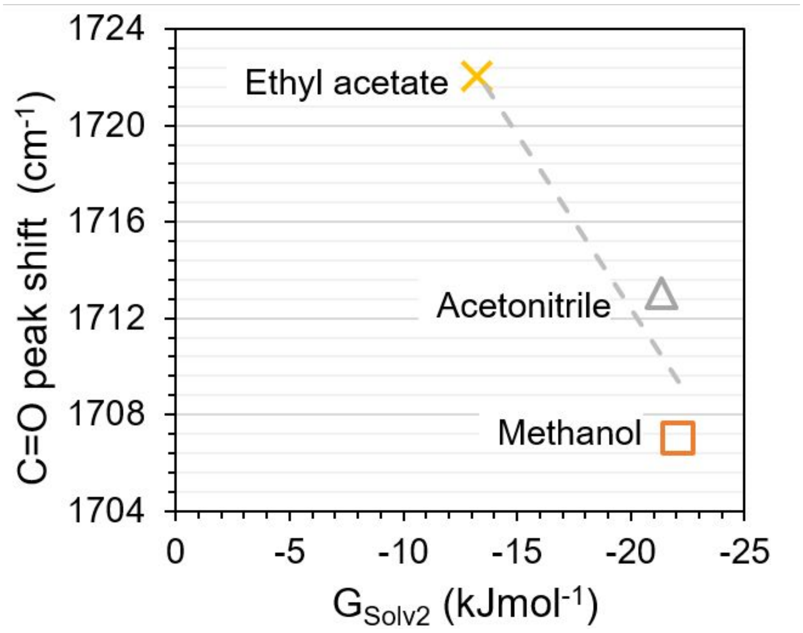

Figure S6. Shift in carbonyl stretching peak *vs.* the solvation free energy values obtained using the methods described above in the three solvents, methanol (orange, □), acetonitrile (grey, Δ) and ethyl acetate (yellow, ×).

Supplementary References

(1) Vanommeslaeghe, K.; MacKerell Jr, A. D. Automation of the CHARMM General Force Field (CGenFF) I: Bond Perception and Atom Typing. *J Chem Inf Model* **2012**, *52* (12), 3144–3154.

(2) Vanommeslaeghe, K.; Raman, E. P.; MacKerell Jr, A. D. Automation of the CHARMM General Force Field (CGenFF) II: Assignment of Bonded Parameters and Partial Atomic Charges. *J Chem Inf Model* **2012**, *52* (12), 3155–3168.

(3) Groom, C. R.; Bruno, I. J.; Lightfoot, M. P.; Ward, S. C. The Cambridge Structural Database. *Acta Crystallogr B Struct Sci Cryst Eng Mater* **2016**, *72* (2), 171–179.

(4) Phetmung, H.; Musikapong, K.; Srichana, T. Thermal Analysis, Structure, Spectroscopy and DFT Calculations of a Pharmaceutical Cocrystal of Salicylic Acid and Salicylamide. *J Therm Anal Calorim* **2019**, *138*, 1207–1220.

(5) Srivastava, R. Application of Optimization Algorithms in Clusters. *Front Chem* **2021**, *9*, 637286.

(6) Gale, J. D. GULP: A Computer Program for the Symmetry-Adapted Simulation of Solids. *Journal of the Chemical Society, Faraday Transactions* **1997**, *93* (4), 629–637.

(7) Huang, J.; Rauscher, S.; Nawrocki, G.; Ran, T.; Feig, M.; de Groot, B. L.; Grubmüller, H.; MacKerell, A. D. CHARMM36m: An Improved Force Field for Folded and Intrinsically Disordered Proteins. *Nat Methods* **2017**, *14* (1), 71–73.

(8) Gale, J. D. Empirical Potential Derivation for Ionic Materials. *Philosophical Magazine B* **1996**, *73* (1), 3–19.

(9) Duarte Ramos Matos, G.; Kyu, D. Y.; Loeffler, H. H.; Chodera, J. D.; Shirts, M. R.; Mobley, D. L. Approaches for Calculating Solvation Free Energies and Enthalpies Demonstrated with an Update of the FreeSolv Database. *J Chem Eng Data* **2017**, *62* (5), 1559–1569. https://doi.org/10.1021/acs.jced.7b00104.

(10) Shirts, M. R. Best Practices in Free Energy Calculations for Drug Design. In *Methods in Molecular Biology*; Springer, 2012; Vol. 819, pp 425–467. https://doi.org/10.1007/978-1-61779-465-0_26.

(11) Sun, H.; Jin, Z.; Yang, C.; Akkermans, R. L. C.; Robertson, S. H.; Spenley, N. A.; Miller, S.; Todd, S. M. COMPASS II: Extended Coverage for Polymer and Drug-like Molecule Databases. *J Mol Model* **2016**, *22* (2), 1–10. https://doi.org/10.1007/s00894-016-2909-0.

(12) Nauchitel, V. V. Energy Distribution Function for the NVT Canonical Ensemble. *Mol Phys* **1981**, *42* (5), 1259–1265. https://doi.org/10.1080/00268978100100941.

(13) Frank, J.; Gottwald, G. A. The Langevin Limit of the Nosé-Hoover-Langevin Thermostat. *J Stat Phys* **2011**, *143* (4), 715–724. https://doi.org/10.1007/s10955-011-0203-1.

(14) Abraham, M. J.; Murtola, T.; Schulz, R.; Páll, S.; Smith, J. C.; Hess, B.; Lindahl, E. GROMACS: High Performance Molecular Simulations through Multi-Level Parallelism from Laptops to Supercomputers. *SoftwareX* **2015**, *1*, 19–25.

(15) Van Der Spoel, D.; Lindahl, E.; Hess, B.; Groenhof, G.; Mark, A. E.; Berendsen, H. J. C. GROMACS: Fast, Flexible, and Free. *J Comput Chem* **2005**, *26* (16), 1701–1718.

(16) Xu, L.; Sun, H.; Li, Y.; Wang, J.; Hou, T. Assessing the Performance of MM/PBSA and MM/GBSA Methods. 3. The Impact of Force Fields and Ligand Charge Models. *J Phys Chem B* **2013**, *117* (28), 8408–8421.

(17) Kollman, P. A.; Massova, I.; Reyes, C.; Kuhn, B.; Huo, S.; Chong, L.; Lee, M.; Lee, T.; Duan, Y.; Wang, W. Calculating Structures and Free Energies of Complex Molecules: Combining Molecular Mechanics and Continuum Models. *Acc Chem Res* **2000**, *33* (12), 889–897.

(18) Kumari, R.; Kumar, R.; Lynn, A. G-Mmpbsa -A GROMACS Tool for High-Throughput MM-PBSA Calculations. *J Chem Inf Model* **2014**, *54* (7), 1951–1962. https://doi.org/10.1021/ci500020m.

(19) Clark Still, W.; Tempczyk, A.; Hawley, R. C.; Hendrickson, T. Semianalytical Treatment of Solvation for Molecular Mechanics and Dynamics. *J Am Chem Soc* **1990**, *112* (16), 6127–6129. https://doi.org/10.1021/ja00172a038.

(20) Honig, B.; Nicholls, A. Classical Electrostatics in Biology and Chemistry. *Science (1979)* **1995**, *268* (5214), 1144–1149. https://doi.org/10.1126/science.7761829.

(21) Srinivasan, J.; Cheatham, T. E.; Cieplak, P.; Kollman, P. A.; Case, D. A. Continuum Solvent Studies of the Stability of DNA, RNA, and Phosphoramidate-DNA Helices. *J Am Chem Soc* **1998**, *120* (37), 9401–9409. https://doi.org/10.1021/ja981844+.

(22) Lide, D. R. *CRC Handbook of Chemistry and Physics*; CRC press, 2004; Vol. 85.
